# High-throughput platform for yeast morphological profiling predicts the targets of bioactive compounds

**DOI:** 10.1038/s41540-022-00212-1

**Published:** 2022-01-27

**Authors:** Shinsuke Ohnuki, Itsuki Ogawa, Kaori Itto-Nakama, Fachuang Lu, Ashish Ranjan, Mehdi Kabbage, Abraham Abera Gebre, Masao Yamashita, Sheena C. Li, Yoko Yashiroda, Satoshi Yoshida, Takeo Usui, Jeff S. Piotrowski, Brenda J. Andrews, Charles Boone, Grant W. Brown, John Ralph, Yoshikazu Ohya

**Affiliations:** 1 Department of Integrated Biosciences, Graduate School of Frontier Sciences, University of Tokyo, Kashiwa, Chiba, 277-8561 Japan; 2 Department of Biochemistry, DOE Great Lakes Bioenergy Research Center and Wisconsin Energy Institute, University of Wisconsin-Madison, Madison, WI 53726 USA; 3 State Key Laboratory of Pulp and Paper Engineering, South China University of Technology, Guangzhou, 510640 China; 4 Departments of Plant Pathology, University of Wisconsin-Madison, Madison, WI 53706 USA; 5 Departments of Plant Pathology, University of Minnesota, St. Paul, MN 55108 USA; 6 Addis Ababa Science and Technology University, College of Biological and Chemical Engineering, P.O. Box 16417, Addis Ababa, Ethiopia; 7 Research Center, Research Division, Nihon Nohyaku Co., Ltd., 345 Oyamada-cho, Kawachi-nagano, Osaka, 586-0094 Japan; 8 RIKEN Center for Sustainable Resource Science, Wako, Saitama, 351-0198 Japan; 9 School of International Liberal Studies, Waseda University, Shinjuku-ku, Tokyo, 169-8050 Japan; 10 Faculty of Life and Environmental Sciences, University of Tsukuba, 1-1-1 Tennodai, Tsukuba, 3058572 Japan; 11 Yumanity Therapeutics, Cambridge, MA 02139 USA; 12 Donnelly Centre for Cellular and Biomolecular Research, University of Toronto, Toronto, ON M5S 3E1 Canada; 13 Department of Biochemistry, University of Toronto, 1 King’s College Circle, Toronto, ON M5S 3E1 Canada; 14 AIST-UTokyo Advanced Operando-Measurement Technology Open Innovation Laboratory (OPERANDO-OIL), National Institute of Advanced Industrial Science and Technology (AIST), Kashiwa, Chiba, 277-8565 Japan; 15 Collaborative Research Institute for Innovative Microbiology, The University of Tokyo, 1-1-1 Yayoi, Bunkyo-ku, Tokyo, 113-8657 Japan

**Keywords:** Target identification, Single-cell imaging, Screening, Antimicrobials, Cell biology

## Abstract

Morphological profiling is an omics-based approach for predicting intracellular targets of chemical compounds in which the dose-dependent morphological changes induced by the compound are systematically compared to the morphological changes in gene-deleted cells. In this study, we developed a reliable high-throughput (HT) platform for yeast morphological profiling using drug-hypersensitive strains to minimize compound use, HT microscopy to speed up data generation and analysis, and a generalized linear model to predict targets with high reliability. We first conducted a proof-of-concept study using six compounds with known targets: bortezomib, hydroxyurea, methyl methanesulfonate, benomyl, tunicamycin, and echinocandin B. Then we applied our platform to predict the mechanism of action of a novel diferulate-derived compound, poacidiene. Morphological profiling of poacidiene implied that it affects the DNA damage response, which genetic analysis confirmed. Furthermore, we found that poacidiene inhibits the growth of phytopathogenic fungi, implying applications as an effective antifungal agent. Thus, our platform is a new whole-cell target prediction tool for drug discovery.

## Introduction

Target identification and validation of specific compounds are crucial for drug discovery and development. During the development of therapeutic and diagnostic drugs, two strategies have been used: target-based drug discovery and phenotypic drug discovery^1^. In target-based drug discovery, the target must be known in advance and is used to screen compounds in biochemical assays. In phenotypic drug discovery, the target may be unknown, and the screened compounds must subsequently be analyzed in detail to uncover the affected targets^1,2^. As a result, strategies for target deconvolution of the compounds found are necessary to elucidate the underlying mechanisms of action^3^.

The budding yeast *Saccharomyces cerevisiae* has been used for target identification of many bioactive compounds^4,5^. In addition to biochemical assays used to evaluate the effects of compounds on the enzymes expressed in yeast^6^, several omics-based approaches have provided useful information on the targets of various compounds. One of the first unbiased approaches was based on gene-expression profiling and comparison of a genome expression profile after drug treatment with a compendium of gene expression profiles derived from gene-deleted cells^7^. It has recently become possible to reveal the functions of new compounds more effectively by combining information other than gene expression^8^. A widely employed method is based on chemical–genetic profiling to predict targets from data; this method comprehensively explores which yeast mutant strains show drug-sensitive or resistant phenotypes with barcoded strains^4,9^. By comparing the chemical–genetic profiling data with a comprehensive genetic interaction database^10^, it is possible to statistically ascertain, which gene activity is inhibited and anticipate annotation of the target function^11^. By comparing chemical–genomic profiles using different compounds, it is also possible to identify compounds with the same mechanism of action^12^. Another method uses morphological profiling and compares the dose-dependent morphological changes induced by drug treatment with the morphological changes in gene-deleted cells^13^. Morphological profiling hypothesizes that a gene deletion with high morphological similarity to the drug-induced changes is likely to be defective in the activity targeted by the compound. Thus, budding yeast provides a target identification system with a unique omics approach. Drug target prediction using morphological profiling was initially developed in 2010^14^. Using this technique, more than ten compounds with known targets and two with unknown targets were predicted and the predictions proved to be correct^15–18^. Among the predicted compounds without known targets, poacic acid is a newly discovered compound in lignocellulosic hydrolysates of grasses^18^. As it is an effective antifungal agent against phytopathogenic fungi such as the filamentous fungi *Sclerotinia sclerotiorum* and *Alternaria solani*, as well as the oomycete *Phytophthora sojae*, it has attracted attention as an agrochemical candidate^3,19,20^. Morphological profiling revealed that cells treated with poacic acid were significantly similar to *fks1∆*, which harbors a deletion of the gene encoding the catalytic subunit of 1,3-β-glucan synthase. It was confirmed in vitro and in vivo that poacic acid binds to 1,3-β-glucan, which is the main component of the fungal cell wall, leading to a decrease in 1,3-β-glucan synthase activity. However, the conventional morphological profiling method has several limitations: it requires high concentrations of the compounds, the experiment is time-consuming, and it is necessary to extract reliable information from noisy morphological data.

In this study, we developed a new and reliable high-throughput (HT) platform for morphological profiling that is designed to predict the targets of bioactive compounds in yeast cells. For this purpose, we used a diagnostic set of viable yeast gene-deletion mutants that exhibit significant morphological phenotypes within a drug-hypersensitive strain. We also employed an automatic HT microscope coupled with the CalMorph image-processing system specialized for yeast morphology^21^. Finally, we applied basic machine-learning techniques with a statistical model to predict specific biological functions affected by the compounds. After conducting a proof-of-concept study, we applied this platform to predict the mechanism of action of a novel diferulate compound, poacidiene. We confirmed that poacidiene affects the DNA damage response and inhibits the growth of phytopathogenic fungi, indicating that poacidiene is a novel antifungal agent with a new mechanism of action.

## Results

### Overview of the screening platform

**Fig. 1:**
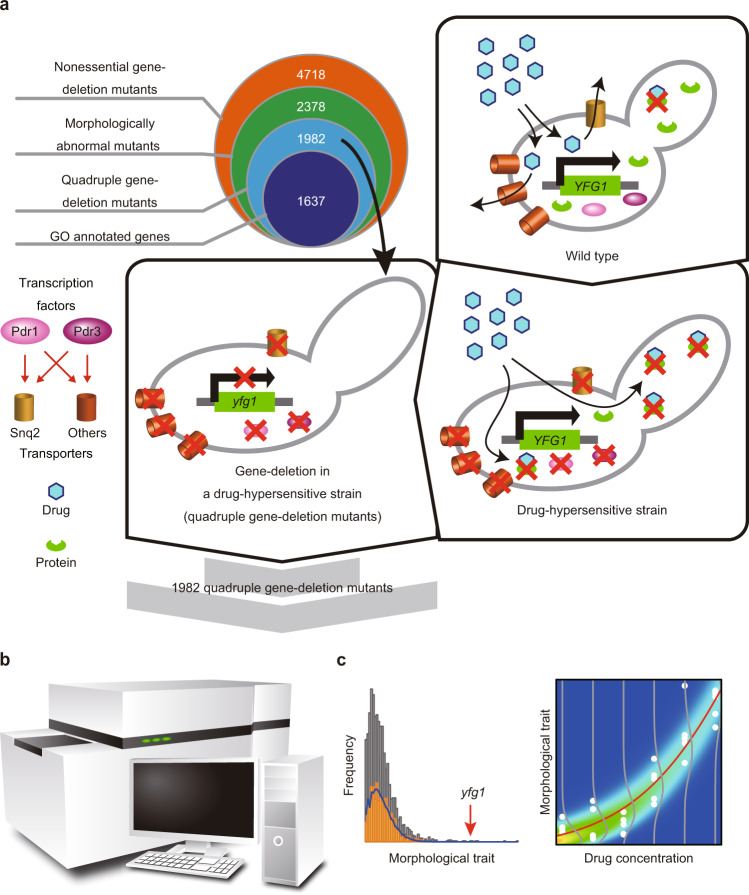
Schematic illustration of the new platform for morphological profiling in yeast. **a** Selection of genes deleted in a drug-hypersensitive strain. Haploid mutant strains in which each gene was individually deleted in a drug-hypersensitive strain with a triple-deletion genetic background were constructed for 1637 non-essential genes with GO annotation and used for the morphological profiling and functional annotation of chemical compounds (blue circle in top left panel). Of the 4718 non-essential genes in the yeast genome (orange circle in top left panel), 2378 genes (green circle in top left panel) were selected as morphologically important genes. Among 1982 quadruple mutants (cyan circle in top left panel) successfully constructed by gene deletion in a drug-hypersensitive strain (3∆; *pdr1∆ pdr3∆ snq2∆*, middle panel), 1637 genes had GO annotation. **b** High-throughput microscopy system coupled with the image-processing system CalMorph. Fluorescence microscope images of the yeast quadruple deletion collection and drug-treated yeast cells were acquired by HT microscopy and subjected to the image-processing program CalMorph (bottom left panel) to quantify morphological features of 501 traits. **c** Application of a generalized linear model to predict biological processes targeted by the bioactive compounds. Morphological data of the quadruple mutants and drug-treated cells were normalized and subjected to analysis with a GLM to predict biological processes targeted by the bioactive compounds (bottom right panel).

To design a new platform for morphological profiling and functional annotation of chemical compounds (Fig. 1), we first selected 2378 genes important for yeast morphology from 4718 non-essential genes and successfully constructed 1982 haploid mutant strains in which each gene was individually deleted in a drug-hypersensitive strain with a triple-deletion genetic background (Fig. 1a). Second, we used an automatic HT microscope to take images of yeast cells after triple staining of the cell wall, actin, and nuclear DNA (Fig. 1b). The images were then processed using the image-processing system CalMorph^21^. Third, we applied a generalized linear model (GLM) to statistically compare the morphological profiles (i.e., profiles of morphological signatures) of chemical-treated cells with those of yeast gene-deletion mutants and predict biological processes targeted by the chemical compounds (Fig. 1c). The basic concept of morphological profiling is that inhibition of the protein activity targeted by the chemical compound results in morphological phenotypes similar to the deletion mutant of the target gene. The effects of the chemical compounds were assessed by detecting dose-dependent morphological changes. Morphological similarity between chemically treated cells and gene-deleted cells was then examined by calculating the correlation coefficients of PC scores.

### Morphological characteristics of the drug-hypersensitive yeast strain

**Fig. 2:**
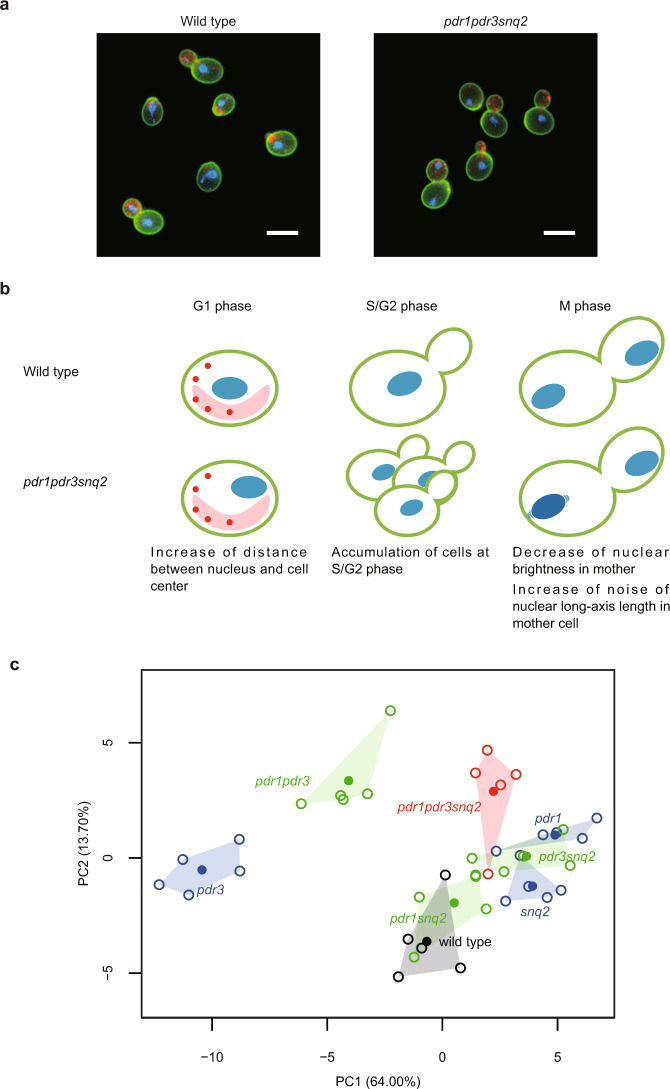
Morphological phenotype of *pdr1 pdr3 snq2*. **a** Cells of a drug-hypersensitive 3∆ strain and its parental strain. The cell wall (green), nuclear DNA (blue), and actin cytoskeleton (red) were stained with FITC-ConA, DAPI, and rhodamine-phalloidin, respectively. Scale bars indicate 5 μm. **b** Illustration of the morphological phenotype of 3∆. Color legend is the same as (**a**). **c** PCA plot showing the mutual relationship of 3∆ (red), 2∆ (green), 1∆ (blue), and their parental strain (black). Open and closed circles indicate biological replicates (*n* = 5) and mean of each strain.

**Fig. 3:**
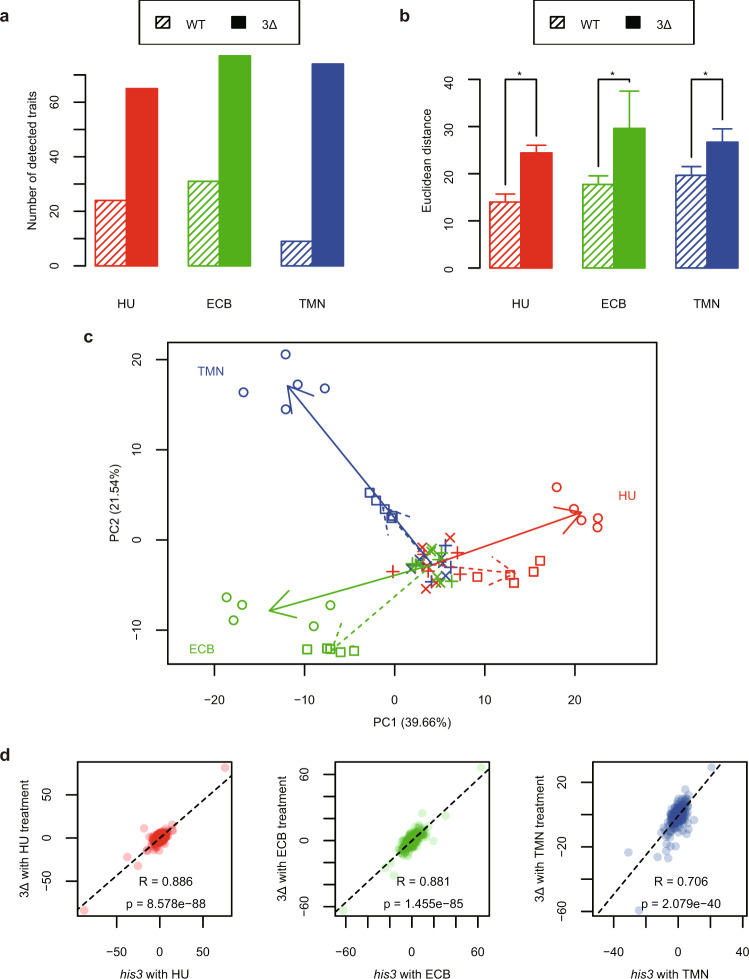
Effectiveness of bioactive compounds in the drug-hypersensitive strain. **a** Number of morphological traits changed by drug treatment in the drug-hypersensitive strain (solid bar) and its wild-type strain (*his3Δ*, shaded bar). HU, ECB, and TMN indicate 8 and 10 mM of HU for the drug-hypersensitive strain and *his3Δ*, respectively, 2.0 μg/mL ECB for both strains, and 100 ng/mL TMN for both strains. **b** Drug-treatment-induced changes in holistic morphological abnormality in the drug-hypersensitive strain (solid bar) and *his3Δ* (shaded bar). Error bars indicate standard deviations. Asterisks indicate a significant difference at *p* < 0.01 after Bonferroni correction by the likelihood-ratio test. **c** PCA plot showing morphological profiles of the drug-hypersensitive strain (circle) and *his3Δ* (square) induced by HU, ECB, and TMN. The drug-hypersensitive strain (cross) and *his3Δ* (plus) without drug treatment are also shown. Directions of morphological changes induced by HU, ECB, and TMN are shown as arrows in the drug-hypersensitive cells (solid) and *his3Δ* (dashed). Percentages in parentheses indicate the proportion of variance explained by each PC. **d** Similarity of morphological profiles between the drug-hypersensitive strain and *his3Δ* after treatment with HU, ECB, and TMN. PC scores of 259 PCs were plotted between *his3Δ* (*x*-axis) and 3∆ (*y*-axis) after chemical treatment. *R* and *p* indicate the Pearson product-moment correlation coefficient and the *p*-value calculated by the t-distribution.

To increase the accessibility of the bioactive compounds in yeast cells, we used a drug-hypersensitive strain with a triple-deletion genetic background, as described by Piotrowski et al.^11^. Briefly, this strain was constructed by combining deletions of *PDR1* and *PDR3*, both of which encode transcription factors that regulate the yeast pleiotropic drug response, with a deletion of *SNQ2*, which encodes a multidrug transporter. After staining the cell wall, actin, and nuclear DNA, we first compared the morphology of the drug-hypersensitive strain 3∆ (*pdr1∆ pdr3∆ snq2∆*) with that of its parental wild-type strain incubated in YPD medium (Fig. 2a). We found that morphological features were altered in the drug-hypersensitive strain, including nuclear morphology, subcellular arrangement of the nucleus, and accumulation of S/G2-phase cells (Fig. 2b). Among 501 morphological traits, 16 were significantly changed (Wald test, false discovery rate [FDR] = 0.02). After principal component analysis (PCA), morphological phenotypes of single-, double-, and triple-deletion mutant cells were mapped in the two-dimensional principal component space (hereafter called the morphological space). Figure 2c illustrates that the morphology of single mutants of *pdr1∆, pdr3∆*, and *snq2∆* differed from the parental strain and that the morphology of the triple mutant also differed from each single mutant, exhibiting a characteristic morphology (Fig. 2b). To determine whether bioactive compounds had more profound effects on morphology in the drug-hypersensitive strain than in its parental strain, we compared the extent of morphological changes after treatment with three well-known chemical compounds (HU, ECB, and TMN). Each compound induced more morphological changes in the drug-hypersensitive strain with an increased number of altered parameters (Fig. 3a). The extent of morphological abnormality was calculated using Euclidean distance, as described in Suzuki et al. (2018)^22^. Briefly, the Euclidean distance was calculated from untreated to treated samples in an orthogonal six-dimensional space. We found that the holistic morphological abnormality was significantly increased in the drug-hypersensitive strain (Fig. 3b; likelihood ratio test, *p* < 0.01 after Bonferroni correction). Although the morphological abnormality induced by each chemical compound increased, the direction of morphological changes did not change in the morphological space (Fig. 3c). The morphological similarity between the drug-hypersensitive strain and its parent was quantitatively analyzed by calculating the correlation coefficients of principal component (PC) scores. We used the PC scores of 259 PCs covering a cumulative contribution of 99% (see “Materials and Methods”). High correlation coefficients were observed after treatment with each chemical compound (non-correlation test, *p* < 0.05 after Bonferroni correction; Fig. 3d), implying that chemically induced morphological changes were conserved in the drug-hypersensitive strain. This implies that a lower concentration of the compounds will still yield many relevant morphological phenotypes in 3∆, demonstrating the advantages of using a drug-hypersensitive strain in morphological profiling experiments.

### Morphological profiling of chemical compounds with known targets

To prove the concept of morphological profiling using the developed dataset, we employed six well-known chemical compounds effective in budding yeast: BTZ (proteasome inhibitor), HU (ribonucleotide reductase inhibitor), MMS (alkylating agent), BML (microtubule-destabilizing drug), TMN (protein glycosylation inhibitor), and ECB (1,3-β-glucan synthase inhibitor).

**Fig. 4:**
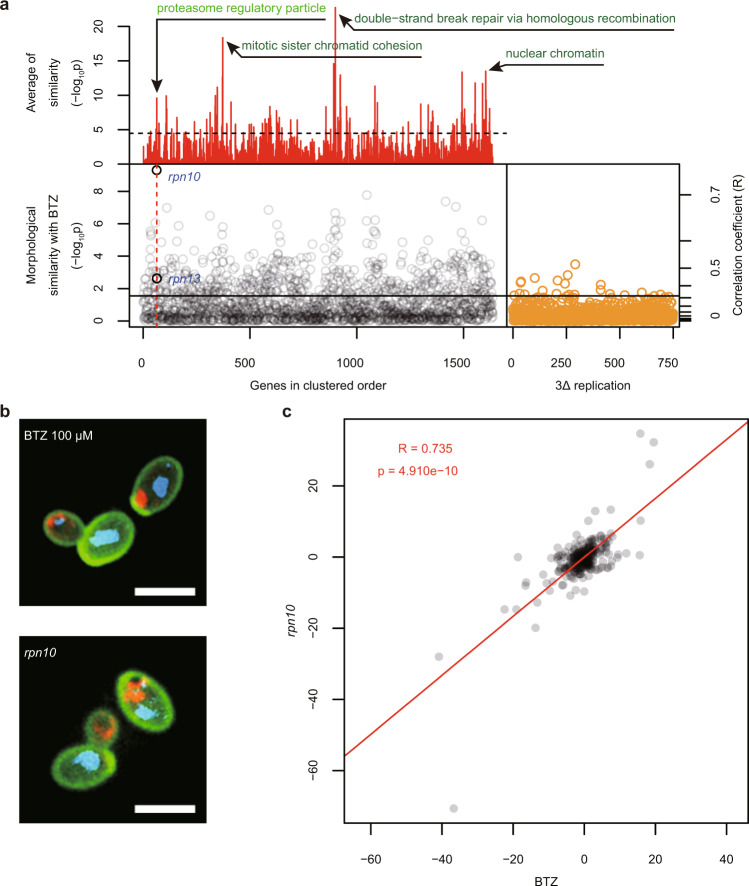
Morphological profiling of bortezomib-treated cells. **a** BTZ-treated cells exhibited the most similar morphology to *rpn10Δ*. The *RPN10* gene belongs to the significantly enriched functional gene group represented as “proteasome regulatory particle (green text)”. The red lines in the top panel represent 1486 groups of the functionally related genes, which are ordered by the similarity of gene functions. Briefly, the distance between genes was calculated using a Boolean matrix with genes as rows and GO as columns, hierarchical cluster analysis was performed based on the obtained distance matrix, and the gene-deleted strains were arranged in the order of the tree diagram of this cluster analysis (see “Materials and Methods”). The *y*-axis indicates average similarities of functionally related genes to BTZ-treated cells as expressed by −log10p (likelihood ratio test). Dark green texts indicate the GO terms enriched in the significant top three groups of the lowest *p* value by the likelihood ratio test among the detected gene groups. The horizontal dashed line indicates a threshold *p*-value of 0.05 after Bonferroni correction. Enriched GOs in the detected gene groups (*p* < 0.05 after the Bonferroni correction) are listed in Supplementary Data 2. The lower panel shows the most similar mutant and the mutant belonging to the functionally related gene shown in the top panel (black), 1635 quadruple mutants (grey), and 749 replicates of 3Δ (orange, ordered by the date of data acquisition). The morphological similarity (*y*-axis) is represented by −log10p, which was calculated by Gaussian distribution fitted to the t-distribution of correlation coefficients from the wild type (*n* = 749). The horizontal solid line indicates threshold *p*-values at FDR = 0.05. The morphological similarity was calculated using five independent biological experiments with bortezomib-treated cells. Significantly similar mutants (FDR = 0.05) are listed in Supplementary Data 1. **b** Triple-staining images of cells of 3Δ treated with BTZ and *rpn10Δ* in the 3Δ background (*rpn10*). The cell wall (green), nuclear DNA (blue), and actin cytoskeleton (red) are shown. Scale bars indicate 5 μm. **c** Correlation coefficient of morphological profiles between BTZ-treated cells and *rpn10Δ*. PC scores of 259 PCs were plotted between BTZ-treated cells (*x*-axis) and *rpn10Δ* (*y*-axis) after chemical treatment. *R*, *p*; the redacid dilactone was made from ferulic acid by line indicates the Pearson product-moment correlation coefficient, *p*-value calculated by the t-distribution of 3Δ replication (*n* = 749), and the linear regression line, respectively.

Comparison of the morphological profiles revealed that BTZ-treated cells were significantly similar to 583 gene-deletion mutants (Supplementary Data 1, one-sample upper-tailed test by Gaussian distribution, FDR = 0.05) and most similar in morphology to a deletion of *RPN10* (a gene encoding the 26S proteasome regulatory subunit) among the 1637 non-essential gene deletion mutants analyzed (Fig. 4a). The correlation coefficient between BTZ-treated cells and *rpn10∆* (Fig. 4b) was 0.735 (one-sample upper-tailed test by Gaussian distribution, *p* = 4.910e^−10^) (Fig. 4c). *RPN10* belongs to the “proteasome regulatory particle” category, one of the 1486 representative functional gene groups that we used for the gene-enrichment analyses (see “Materials and Methods”). The deletion mutants of “proteasome regulatory particles” were also significantly similar (likelihood ratio test, *p* = 3.72e^−7^ after Bonferroni correction; Supplementary Data 2). These results indicate that the cellular functions affected by BTZ can be inferred by comparing the morphological changes induced by the compound with the morphologies of yeast gene-deletion mutants.

Likewise, cells treated with HU were most similar to a deletion of *RRM3*, which encodes a 5′-to-3′ DNA helicase that affects multiple cellular DNA replication and repair processes (Supplementary Fig. 1a and Supplementary Data 3). Cells treated with MMS were most similar to a deletion of *BRE1*, which encodes a ubiquitin-protein ligase involved in double-strand break repair via homologous recombination (Supplementary Fig. 1b and Supplementary Data 4). Cells treated with BML were most similar to a deletion of *CIN4*, which encodes a GTP-binding protein involved in β-tubulin folding (Supplementary Fig. 1c and Supplementary Data 5). Cells treated with TMN were most similar to a deletion of *KEX2*, required for secreted proteins, and more importantly, they were third-most similar to a deletion of *HOC1*, which encodes α-1,6-mannosyltransferase involved in cell-wall mannan protein biosynthesis (Supplementary Fig. 1d and Supplementary Data 6). The most similar mutant to the cells treated with ECB was not a deletion of *FKS1*, which encodes a catalytic subunit of 1,3-β-glucan synthase, probably due to the existence of a functionally redundant gene, *FKS2*. Instead, ECB-treated cells were most similar to a deletion of *MNN10*, which encodes a subunit of the Golgi mannosyltransferase complex involved in cell wall assembly (Supplementary Fig. 1e and Supplementary Data 7).

Gene enrichment analysis revealed that the deletion mutants of the “double-strand break repair” category were significantly similar to the cells treated with HU and MMS (likelihood ratio test, *p* = 8.78e^−11^ and *p* = 9.90e^−10^ after Bonferroni correction, respectively; Supplementary Fig. 1a–b and Supplementary Data 8–9). In addition, the deletion mutants of “tubulin complex assembly,” “protein glycosylation,” and “α-1,6-mannosyltransferase activity involved in cell wall synthesis” were significantly similar to the cells treated with BML, TMN, and ECB, respectively (likelihood ratio test, *p* = 5.55e^−4^, *p* = 1.71e^−4^, and *p* = 3.61e^−15^ after Bonferroni correction, respectively; Supplementary Fig. 1c–e and Supplementary Data 10–12). Taken together, we concluded that morphological profiling can be used to predict the cellular functions affected by diverse chemical compounds.

### Comparison with the previous database

**Fig. 5:**
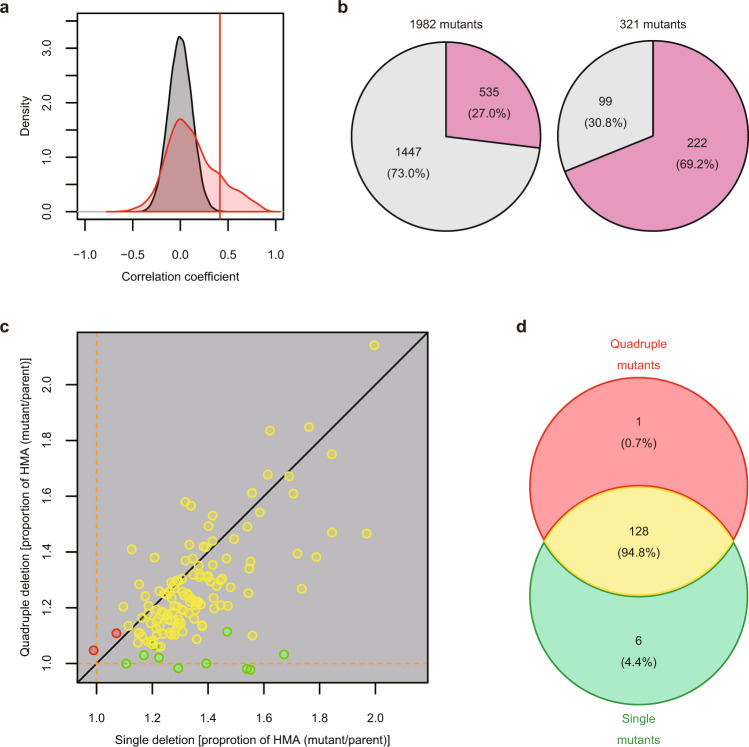
Reproducibility of morphological phenotypes. **a** Distribution of correlation coefficients between quadruple mutants and single mutants. Correlation coefficients were calculated between each pair of the 1982 deletion mutants in quadruple mutants and single mutants (red) and between arbitrary pairs of 3∆ (*n* = 749) and *his3∆* (*n* = 109) (grey), and are expressed using density plots. Vertical red lines indicate a threshold at FDR = 0.05 by the one-tailed t-distribution test. **b** Percentages of gene-deletion mutants showing reproducibility. Among 1982 common gene-deleted strains (left), 27.0% were detected as having significantly similar morphological phenotypes (FDR = 0.05 by the one-tailed t-distribution test) between single and quadruple mutants. Among 321 quadruple mutants with significantly high HMA, 69.2% were detected as having significant phenotypic similarity (FDR = 0.05 by the one-tailed t-distribution test). **c** Comparison of average HMA in the genes belonging to the same gene function groups. Orange dashed lines indicate averages of the parental strains. Black line indicates the same HMA between quadruple and single mutants. Green and red circles indicate the gene groups with significantly high average HMA in the single- and quadruple-deletion mutants (Wald test, FDR = 0.05), respectively. Yellow circles indicate the gene groups with similar HMA. **d** Venn diagram of the gene groups; the color legend is the same as in (**c**).

The previous morphological dataset was obtained by examination of a BY4741-derived haploid mutant strain collection in which each non-essential gene was individually deleted^21^. This dataset (4718 gene-deletion mutants) has been successfully used for drug target prediction^15,16,18^. To determine whether the current dataset using the drug-hypersensitive strain is as reliable as the previous dataset, we tested the morphological similarity of the deletion mutants between single- and quadruple-deleted strains. The distribution of the correlation coefficients (Fig. 5a) revealed that among 1982 common gene-deleted strains, only 27.0% were significantly similar (one-sample upper-tailed test by Gaussian distribution, FDR = 0.05; Fig. 5b and Supplementary Data 13). The deletion mutants with high holistic morphological abnormality (HMA) tended to show distinct and characteristic morphological phenotypes, while those with low HMA showed morphologies indistinguishable from noise^22^. As a result, among 321 deletion mutants with significantly high HMA (one-sample upper-tailed test by Gamma distribution, FDR = 0.05), significantly similar mutants (one-sample upper-tailed test by Gaussian distribution, FDR = 0.05) increased to 69.2% (Fig. 5b and Supplementary Data 13). We then compared HMAs of single-deleted strains and quadruple-deleted strains. HMAs of the genes in 128 representative functional gene groups were plotted almost equally near the *y* = *x* line (Fig. 5c, yellow), with only six single-deleted strains showing higher HMAs (Fig. 5c, green). A Venn diagram indicated that the average HMAs of the strains defective in 94.8% of gene groups were quite similar (Fig. 5d). This implies that high-HMA genes are reliably observed in the quadruple-deleted strains, indicative of definitive morphological data. These results imply that our newly developed morphological database is sufficiently reliable to evaluate the morphological similarity of deletion mutants.

### Morphological profiling of a chemical compound with an unknown target

**Fig. 6:**
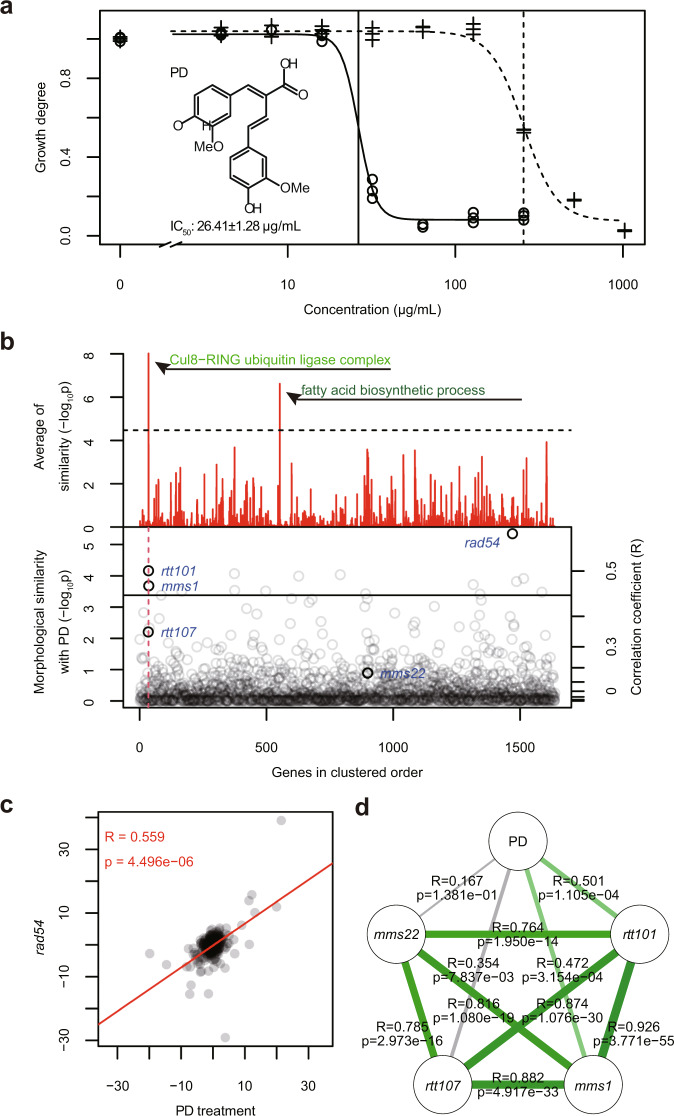
Prediction of the intracellular target of poacidiene. **a** Growth inhibition of yeast strain (3∆) by poacidiene (PD) and poacic acid (PA). The solid curve with circles and the dashed curve with plus symbols indicate approximated logistic curves of PD and PA, respectively. Vertical lines indicate IC_50_ values estimated by logistic regression (PD: 26.41 ± 1.28 μg/mL, PA: 255.2 ± 7.8 µg/mL, mean ± standard error). **b** Morphological similarity of PD-treated cells with 1637 quadruple mutants. Legends are the same as Fig. 4a. Enriched GOs in the detected gene groups (*p* < 0.05 after the Bonferroni correction by likelihood ratio test) are listed in Supplementary Data 15. Significantly similar mutants (FDR = 0.05 by one-sample test with Gaussian distribution fitted to the t-distribution of correlation coefficients from the wild type) are listed in Supplementary Data 14. **c** Similarity of morphological profiles between the PD-treated cells and *rad54∆*. Legends are the same as Fig. 4c. **d** Similarity of the morphological profiles among PD-treated cells (PD) and Cul8-RING gene-deletion mutants. Green lines (but not grey) indicate significantly high correlation coefficients at FDR = 0.05. *R* and *p* indicate the Pearson product-moment correlation coefficient and *p*-value calculated by one-tailed t-distribution, respectively.

We next attempted to predict cellular pathways affected by a chemical compound with unknown targets. We previously screened a collection of diferulates and their derived compounds present in lignocellulosic hydrolysates of grasses for antifungal activity and found poacic acid to be a candidate antifungal agent^18^. Poacic acid binds to 1,3-β-glucan, resulting in a decrease in 1,3-β-glucan synthase activity, inhibiting the growth of many plant pathogenic fungi. Further screening of diferulates led us to identify a new compound, (2*E*,3*E*)-4-(4-hydroxy-3-methoxyphenyl)-2-[(4-hydroxy-3-methoxyphenyl)methylidene]but-3-enoic acid (hereafter named poacidiene) as a more potent antifungal agent. Poacidiene had greater antifungal activity than poacic acid (Fig. 6a), with a half-maximal inhibitory concentration (IC_50_) of 26.4 μg/mL against our control yeast (3∆).

Morphological profiling with the current platform revealed that poacidiene-treated cells were significantly similar to 17 deletion mutants (Supplementary Data 14, one-sample upper-tailed test by Gaussian distribution, FDR = 0.05) and most similar in morphology to a deletion of *RAD54* (a gene involved in recombinational repair of double-strand breaks) among the 1637 non-essential gene-deletion mutants analyzed (Fig. 6b). The correlation coefficient between poacidiene-treated cells and *rad54∆* was 0.559 (one-sample upper-tailed test by Gaussian distribution, *p* = 4.496e^−9^) (Fig. 6c). By contrast, the correlation coefficient between profiles of poacidiene-treated cells and those of poacic acid-treated cells was 0.15, showing no significant morphological similarity at p = 0.05. Gene enrichment analysis revealed that the deletion mutants in the “Cul8-RING ubiquitin ligase complex” category were significantly similar to poacidiene-treated cells (likelihood ratio test, *p* = 1.427e^−^^5^ after the Bonferroni correction) (Supplementary Data 15). Among four genes involved in the Cul8-RING ubiquitin ligase complex, deletions of *RTT101* and *MMS1* were significantly similar to poacidiene-treated cells (Fig. 6d). Cul8-RING ubiquitin ligase complex is also involved in replication repair^22^, implying that poacidiene affects cellular pathways related to DNA damage response.

### Genetic and mycotic studies on poacidiene

**Fig. 7:**
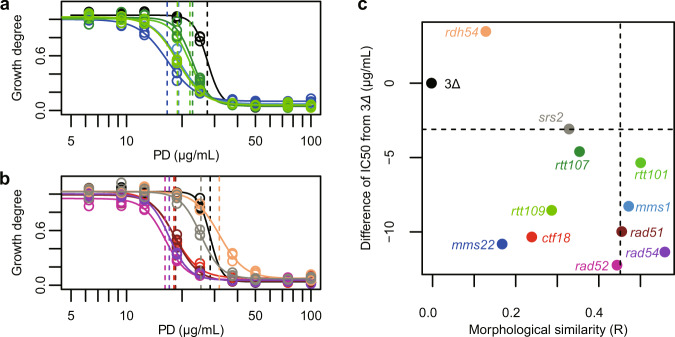
Sensitivity of poacidiene in several gene-deletion mutants defective in the recombinational repair pathway. Inhibitory effects of poacidiene (PD) for cell growth in mutants of **a** Cul8-RING and **b** the recombinational repair pathway. Vertical lines indicate estimated IC_50_ values. Colors correspond to strains indicated in (**c**). **c** Distributions of morphological similarity and IC_50_ values. The dashed lines represent significantly different lines compared to 3∆ (*p* < 0.05, likelihood ratio test after Bonferroni correction).

To investigate genetically whether the mechanism of action of poacidiene is related to the recombinational repair pathway, we examined the sensitivity to poacidiene of several yeast mutants defective in the recombinational repair pathway. The parental strain 3∆ and quadruple mutants (*mms1∆, mms22∆, rtt101∆, rtt107∆, rtt109∆, ctf18∆, rad51∆, rad52∆, rad54∆, rdh54∆*, and *srs2∆*) were tested for poacidiene sensitivity ranging from 0 to 100 μg/mL. We found that all mutants with deletions of genes encoding components of the Cul8-RING ubiquitin ligase complex (*mms1∆, mms22∆, rtt101∆, rtt107∆*) were more sensitive to poacidiene than was the parental control strain 3∆ (Fig. 7a). The most sensitive mutant was *mms22∆* with an IC_50_ of 16.60 μg/mL (Supplementary Data 16). In addition to Cul8-RING ubiquitin ligase complex mutants, a deletion of the gene encoding the upstream component, Rtt109, also showed poacidiene sensitivity with an IC_50_ of 18.87 μg/mL (Supplementary Data 16). Among the deletion mutants defective in the recombinational repair pathway, *rad52∆*, *rad54∆*, *ctf18∆*, and *rad51∆* were all significantly sensitive (likelihood ratio test, *p* < 0.05, after Bonferroni correction), whereas *rdh54∆* and *srs2∆* showed similar sensitivity to the parental strain (Fig. 7b). The most morphologically similar mutant, *rad54∆*, was very sensitive to poacidiene (Fig. 7c). However, there was no obvious correlation between morphological similarity and poacidiene sensitivity (Spearman’s rank correlation *R* = −0.350, *p* = 0.266, non-correlation test). These results clearly indicate that the Cul8-RING ubiquitin ligase complex and recombinational repair pathway are required for poacidiene resistance, implying that the mechanism of action of poacidiene is related to the DNA-damage-response pathway.

**Fig. 8:**
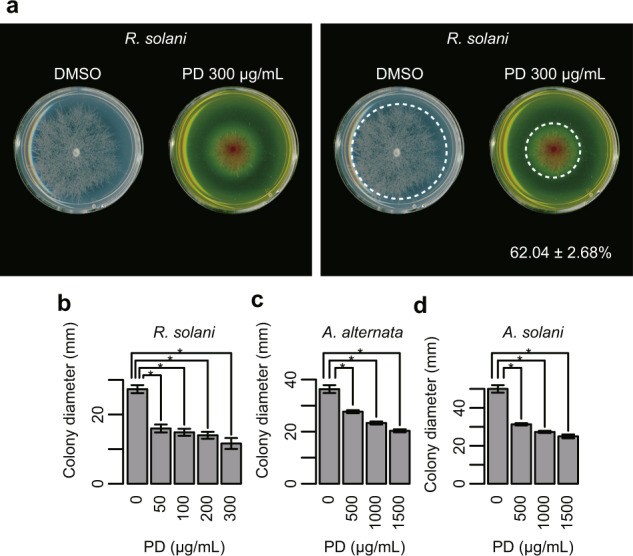
Sensitivity of phytopathogenic filamentous fungi to poacidiene. **a** The effects of poacidiene (PD) on the growth of the filamentous fungi *R. solani* at 300 µg/mL. The radial growth of mycelia was tested (*n* = 3) after 24 h, and PDA plates containing DMSO were used as control plates. The percentage and ± standard error of the growth inhibition by PD (*n* = 3) were estimated by maximum likelihood estimation of one-way ANOVA assuming gamma distribution to colony diameter (mm) compared with the growth on the control plates (*n* = 3). The images with and without a dotted circumference are shown after enhancing the contrast. The raw images are shown in Supplementary Fig. 5. The dotted circle indicates the edge of the colony. The dose-dependent inhibition of **b** *R. solani* after 24 h, **c** *A. alternata* after 120 h, and **d** *A. solani* after 120 h by PD. The radial growth of mycelia was tested (*n* = 3). Asterisks indicate a significant difference at *p* < 0.01 by Dunnett’s test. Error bars in the bar plots indicate standard deviations.

**Fig. 9:**
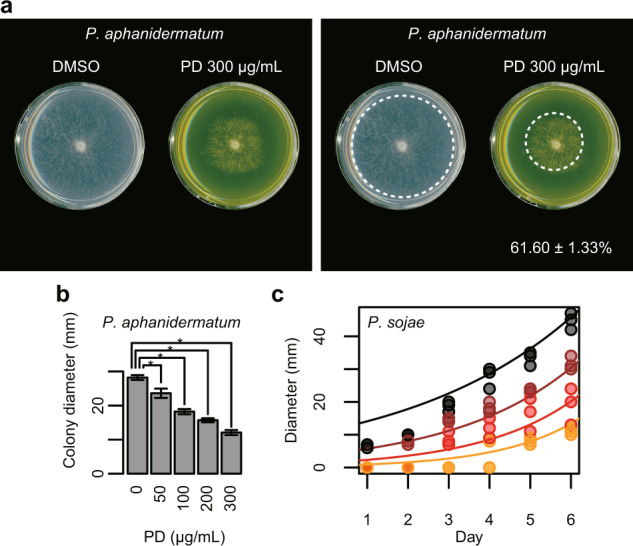
Sensitivity of oomycetes and phytopathogenic fungi to poacidiene. **a** The effects of poacidiene (PD) on the growth of the oomycete *P. aphanidermatum* at 300 µg/mL. The radial growth of mycelia was tested (*n* = 3) after 15 h, and PDA plates containing DMSO were used as control plates. The percentage and ± standard error of the growth inhibition by PD (*n* = 3) was estimated by maximum likelihood estimation of one-way ANOVA assuming gamma distribution to colony diameter compared with the growth on the control plates (*n* = 3). The images with and without a dotted circumference are shown after enhancing the contrast. The raw images are shown in Supplementary Fig. 5. The dose-dependent inhibition of **b** *P. aphanidermatum* after 15 h and **c** *P. sojae* by PD. Asterisks in (**b**) *P. aphanidermatum* indicate a significant difference at *p* < 0.01 by Dunnett’s test. Error bars indicate standard deviations. The regression curve in (**c**) *P. sojae* was estimated by maximum likelihood estimation of multiple linear regression analysis assuming negative binomial distribution to colony diameter (mm, *n* = 72) with the cultivation time (day) and the concentration (µg/mL) of the PD treatment as explanatory variable (*p* = 4.39e^−23^ by likelihood ratio test). Black, brown, red, and orange lines and dots indicate 0, 500, 1000, and 1500 µg/mL of PD.

Poacic acid inhibits the growth of pathogenic fungi, including filamentous fungi and oomycetes^18^. Therefore, we investigated the effects of poacidiene on the growth of the filamentous fungi *Rhizoctonia solani, Alternaria alternata* and *Alternaria solani*, and the oomycetes *Pythium aphanidermatum* and *Phytophthora sojae*. We found that poacidiene inhibited the growth of the filamentous fungi *R. solani* (Fig. 8a, b) at 50 µg/mL as well as *A. alternata* (Fig. 8c) and *A. solani* (Fig. 8d) in a dose-dependent manner. In addition, poacidiene inhibited the growth of the oomycetes *P. aphanidermatum* (Fig. 9a, b) and *P. sojae* (Fig. 9c). Thus, we propose that poacidiene can also be used as an antifungal agent by suppressing the growth of phytopathogenic filamentous fungi and oomycetes.

## Discussion

We developed a new platform for comparing morphological profiles in *S. cerevisiae* using drug-hypersensitive strains to reduce the amounts of chemical compounds required, HT microscopy to increase the analytical speed significantly, and a GLM to predict intracellular targets with high reliability. First, we demonstrated that drug targets can be well predicted by accurately predicting the targets of six compounds with known targets. We confirmed that the reliability of the new platform is as high as that of the conventional system^15,16,18^, which previously showed high performance. We predicted that a novel diferulate-derived compound, poacidiene, would affect the DNA-damage-response pathway and we obtained supporting genetic evidence. As poacidiene works at a lower concentration than the conventional diferulate derivatives, it may become a lead compound in the design of effective antifungal agents.

### Prediction of intracellular targets by morphological profiling

The developed platform calculates the morphological similarity and lists the gene-deletion strains significantly similar to the compound-treated cells by using 501 unbiased morphological traits. In the case of compounds with known targets such as BTZ, HU, MMS, BML, ECB, and TMN, each predicted gene-deletion strain was defective in the function associated with its target. However, the deletion mutants with the highest similarity to the compound-treated cells do not always lack the relevant function of the intracellular target. The morphologies of different gene-deleted strains may coincidentally resemble each other, and mutant strains with pleiotropic morphological effects tend to give a somewhat high degree of morphological similarity. As a result, we believe that predicting targets from the enrichment of gene functions is more reliable than from the morphological similarity of individual gene-deleted strains. In this study, all compounds with known targets used in the experiment showed significant enrichment of target gene function. It should be noted that it is difficult to narrow down the target functions to only one when enrichment of several gene functions is observed. This is because the high-dimensional morphological phenotypes can be coincidentally similar to each other even when unrelated gene functions are deleted.

### Reliability of the morphological dataset

One key factor in morphology-based target prediction is the reliability of the morphological dataset. High-dimensional morphological data of gene-deletion mutants contain important information on gene function that is frequently buried in experimental errors^22^. It is therefore necessary to distinguish meaningful morphological changes from experimental errors and to accurately compare the morphological profiles. To this end, we first constructed a morphological database using only 1982 non-essential deletion mutants whose morphologies changed significantly. Second, as our HT microscope is automatic, it would allow an accurate and reproducible measurement for morphological features^23^. Experimental errors seen in the current morphological data are in fact smaller (65.8% ± 1.0% in mean ± standard error) than the manually obtained morphological data in most of the traits. Third, GLM was used for normalization of the data in each morphological trait to extract the meaningful morphological changes, after the best probability distribution was defined^24^. Finally, a parametric statistical approach was used to extract the dose-dependent chemically induced morphological changes precisely to compare the morphological profile characteristics of each gene-deleted strain. The morphological profiles obtained in this study were, therefore, more reliable than those of the conventional method due improvements in every step in our platform. Notably, the morphological similarity was more clearly detected when mutants with high HMA were used. We obtained wild-type data 749 times and mutant data once for each mutant. In the future, increasing the number of mutant strain replicates will provide a more reliable morphological dataset by calculating the mean of the multiple replicates.

### Morphological profiling of compound-treated cells

We used dose-dependent morphological changes at 5–6 different concentrations rather than the changes themselves induced by the compounds. This was because a dose-effect is expected in pharmacology^18^, in which a larger morphological change should be observed as the amount of a compound increases. Dose-dependent morphological features were extracted after treating the cells with a dilution series of a relatively low concentration of the compound for 16 h. Time-dependent morphological changes can be scored using a higher concentration of the compound^25^, but this is not suited to our platform. This is because the morphological data for comparison were obtained from gene-deleted strains, whose morphological changes were already in a steady state. By analyzing the steady state rather than the transient changes and using the drug-hypersensitive strain as the background, it became possible to predict the intracellular target with a much lower amount of compound.

### Advantages and limitations of the morphological profiling method

Targets of chemical compounds were predicted based on the correlation coefficient of morphological similarity. This implies that predictions were objectively judged using statistical analyses. The extent of morphological abnormality is an important factor that determines the prediction power. If the dose-dependent morphological changes are large, it becomes easier to search for gene-deleted strains with similar patterns of morphological change. On the other hand, it is impossible to predict the target if the morphology does not change even at the highest concentration of a compound. Another point to note is that our target prediction is limited to one major target. In a case in which multiple targets exist, we must compare the morphological changes in the double-mutant strains instead of the current dataset. Previous studies indicated that many genes impact yeast morphology^26–32^. Genes affecting only morphology but not fitness have also been reported^22^. It is therefore possible to perform morphology-based target-prediction even with non-bioactive compounds that do not affect fitness. Genes affecting fitness have also been reported. Fitness profiling and morphological profiling can therefore be proposed to be complementary. In this study, we demonstrated that it is possible to predict the targets of standard compounds that affect various intracellular functions such as the proteasome, DNA replication, double-strand-break repair, β-tubulin, cell-wall mannan protein biosynthesis, and cell-wall assembly. Morphological profiling is therefore a promising tool for predicting the targets of compounds that affect a wide variety of intracellular gene functions.

### Poacidiene as a useful antifungal agent

Poacidiene is from an 8–8 linked dilactone of ferulic acid^33,34^, with an (*E*,*E*) diene having an *s-cis* configuration. Previous studies on the structure–activity relationship of phenolic compounds revealed that the position and number of double bonds and hydroxy and methoxy groups strongly affect their activity^35,36^. In particular, the hydroxy and methoxy groups are essential functional groups that can act as donors or acceptors of hydrogen bonds, possibly being involved in cell-membrane permeability. To develop more active lead compounds, the *s-cis* configuration of (*E*,*E*) diene and the arrangement of methoxy groups of poacidiene are considered important structures for modification.

Poacidiene inhibits the growth of fungi, including the filamentous fungi *R. solani*, *A. alternata* and *A. solani*, and the oomycetes *P. aphanidermatum* and *P. sojae*. We, therefore, propose that this compound can be used as a broad-spectrum antifungal agent. Various derivatives of diferulate are obtained from grasses, some of which exhibit antifungal activity^18^. We also found that poacidiene affects the DNA-damage-response pathway. The different mechanisms of action of poacidiene (DNA-damage response) and poacic acid (cell wall) imply that diferulates and their derived compounds have diverse physiological activities. As fungi resistant to existing antifungal agents have emerged^19^, new lead compounds for antifungal agents with new mechanisms of action are strongly desired. By investigating other diferulates and related compounds, more candidates with new mechanisms of action may be discovered. Further research will be needed to study the structure–activity relationship of such diferulates, including safety verification.

### Use of this platform in future drug discovery

The current platform predicts intracellular targeted pathways statistically from enrichment analysis of gene functions. Using this platform, we propose to review the compound-screening protocol and the modality of target prediction. As it requires a small amount of compound, it becomes possible to handle compounds that have been neglected due to the difficulty of obtaining them in sufficient quantities, which will lead to the discovery of lead compounds having novel skeletons. As ~20% of all human proteins have been proposed as targets for marketed drugs^37–40^, new approaches to study the remaining druggable proteins are drawing attention^41^. Druggability has decisive meaning for drug-discovery research in determining whether a hit compound can evolve into a lead compound. Given the recent emergence of drug-resistant fungi, the development of new antifungal agents is still desired^19,42^. If utilized in the drug-discovery process of antifungal agents and others, this platform will provide useful information as a new tool for target prediction.

### Application of morphological profiling in other organisms

The current platform predicts drug targets using the relationship between the yeast morphological phenotype and gene function. In recent years, the effects of drugs on morphological phenotypes have been actively examined in cells, from bacteria to humans. Morphological phenotypes of bacteria have been quantitatively analyzed after drug treatments^43^, making it possible to predict the mechanisms of action of drugs based on bacterial cytological profiling^44^. The effects of therapeutic agents on human cell morphologies have also been quantitatively analyzed. For example, the cell morphologies of breast cancer and colorectal cancer have been investigated after treatment with drugs, and used for drug evaluation^45^. Morphological and physiological properties of nerve cells, including axon/dendrite formation and action potential amplitudes of individual neurons, have been collected after drug treatments. Based on this type of information, profiling of drugs that act on nerve cells, such as Parkinson’s drugs, should be effective. However, the blueprints for neural circuits are so complex and poorly understood^46^ that even drugs that have advanced to Phase III clinical trials have not been investigated sufficiently as regards their mechanisms of action^46^. What will become increasingly important in the future is research on the relationship between genotype and cell morphological phenotype. If a platform for nerve cells can be established with a system similar to ours, more useful information will be obtained for degenerative neurological diseases in the future. The domino effects of morphological profiling research related to gene function will be wide reaching and it is expected that they will strongly promote future life science research.

## Materials and methods

### Yeast strains used in this study

The drug-hypersensitive yeast strain Y13206 (3∆; *MATα snq2∆:: KlLEU2 pdr3∆:: KlURA3 pdr1∆:: NATMX can1∆an11:: 2iSp_his5 lyp1∆ his3∆1 leu2∆0 ura3∆0 met15∆ LYS2*) and its parent strain Y8835 (*MATα ura3∆0:: natMX4 can 1∆:: STE2pr-Sp_his5 lyp1∆ his3∆1 leu2∆0 met15∆0 LYS2*) were derived from S288C and are described in Piotrowski et al.^11^. The 1∆ (*snq2∆, pdr3∆*, and *pdr1∆*) and 2∆ (*pdr3∆ pdr1∆, snq2∆ pdr1∆*, and *snq2∆ pdr3∆*) strains were also constructed from Y8835. The quadruple gene-deleted strain lacking the gene important for morphology was prepared from the 3∆ strain^11^. Based on the previous morphological database^21^, 2378 genes that significantly impact morphology (*p* < 0.002 for two-sided Gaussian distribution) were selected among 4718 non-essential genes. It was revealed that 396 gene-deletion mutants were lethal or extremely slow to grow in the 3∆ background. Therefore, morphological data were obtained using the remaining 1982 quadruple mutants (Fig. 1a). *his3∆* (BY4741; *MATa his3∆1 leu2∆0 met15∆0 ura3∆0 YOR202w∆:: kanMX4*) used as a parental strain of the Y13206 were purchased from EUROSCARF (acc. no. Y02458, http://www.euroscarf.de/).

### Yeast cell culture and harvest

To obtain high-quality morphological data of the quadruple mutants, cells in the early logarithmic growth phase should be collected each time. Therefore, 1982 quadruple mutants were divided into 24 groups of 5–238 strains by hierarchical cluster analysis of growth phenotypes^47^. Mutants in each group were cultured simultaneously to allow the same time to collect cells. The quadruple mutants and 3∆ were cultured in the same manner as in Ohnuki and Ohya^48^. Briefly, yeasts were picked from frozen stocks, spread on YPD agar plates [1% (w/v) Bacto Yeast Extract (BD Biosciences, San Jose, CA), 2% (w/v) Bacto Peptone (BD Biosciences), and 2% (w/v) glucose (Wako, City, Japan)] and incubated at 25 °C for 3 days to form colonies. The colonies were then inoculated into 2 mL YPD in a 20 mL glass test tube (Iwaki, Shizuoka, Japan) and pre-cultured in a rotator [RT-50; Tokyo Institute of Technology (TITEC), Saitama, Japan] at 30 rpm and 25 °C for 5 h. Then, cells were incubated in 20 mL YPD in a 200 mL conical flask (Iwaki, Shizuoka, Japan) with shaking in a water tank incubator (LT10-F; TITEC) at 110 rpm and 25 °C for 16 h or more. Finally, early logarithmic growth phase cells (5.35 × 10^6^ ± 1.86 × 10^6^ cells/mL) were collected.

### Drug treatment and cell culture

To evaluate the morphological profiling platform, six agents with known targets (HU [Sigma-Aldrich, St. Louis, MO, USA], ECB [a gift from O. Kondo, Chugai Pharmaceutical, Tokyo, Japan], MMS [Sigma-Aldrich], BML [Sigma-Aldrich], BTZ [Sigma-Aldrich], TMN [Sigma-Aldrich]) and one drug with an unknown target (poacidiene, synthesized in this study) were used. The maximum drug concentration used was that inducing ~10% growth inhibition. Drug treatments were carried out at five to six different concentrations including zero in five independent repetitive experiments. For each concentration, the drug was added to YPD medium at the beginning of the main culture and incubated for 16 h. The concentrations of each drug for Y13206 were as follows: HU: 0, 4, 8, 12, 16, and 20 mM; ECB: 0, 0.5, 1.0, 1.5, 2.0, and 2.5 μg/mL; MMS: 0, 0.0002, 0.0004, 0.0006, 0.0008, and 0.0010% (v/v); BML: 0, 0.6, 1.2, 1.8, 2.4, and 3.0 μg/mL; BTZ: 0, 20, 40, 60, 80, and 100 μM; TMN: 0, 25, 50, 75, 100, and 125 ng/mL; poacidiene: 0, 2, 4, 6, 8, and 10 μg/mL in 0.1% DMSO. The concentrations of each drug for *his3∆* were as follows: HU: 0, 5, 10, 20, and 30 mM; ECB: 0, 1, 2, 3, and 4 μg/mL; TMN: 0, 20, 40, 60, 80, and 100 ng/mL.

### Cell fixation, staining, and image acquisition

Cell fixation and staining procedures were described in Okada et al.^49^ Briefly, 20 mL of yeast cell culture in the early logarithmic phase was mixed with 5 mL of fixation solution [37% formalin (2.5 mL) and 1 M potassium phosphate buffer (pH 6.5, 2.5 mL)] in a 50 mL test tube, and stirred at 25 °C for 30 min. Then, the cells were stained with 200 U/mL rhodamine-phalloidin solution (dissolved in methanol, R415; Invitrogen, Carlsbad, CA), 1 mg/mL fluorescein isothiocyanate (FITC)-conjugated concanavalin A (ConA) solution (Sigma-Aldrich), 33.3 ng/mL 4′,6-diamidino-2-phenylindole (DAPI, Cat. 049-18801; Wako) in glycerol solution [600 μL 10× phosphate-buffered saline (T900; TaKaRa, Kyoto, Japan) mixed with 5.4 mL glycerol for fluorescence microscopy (Cat. 104095; Merck, Darmstadt, Germany)]. Triple-stained yeast cells were observed by HT microscopy (IN Cell Analyzer 2000; Cytiva, Tokyo, Japan). A 384-well glass-bottom plate (Cat. 781091; Greiner, Kremsmünster, Austria) coated with 1 mg/mL ConA solution (Cat. 037-08771; Wako) was used. The stained cells were dispensed into a 384-well plate and centrifuged at 395 × *g* for 5 min using a microcentrifuge (CF7D2; Hitachi Koki, Tokyo, Japan) to bind the cells to the bottom of the plate. A 100× objective lens (Nikon, Tokyo, Japan) and three channels (FITC filter, DAPI filter, and Cy3 filter) were used. More than 200 triple-stained cells were photographed in 49 (7 × 7) different fields.

### Quantification and normalization of cell morphology

The images of triple-stained yeast cells were saved in 8-bit grayscale JPEG format and processed by the image processing program CalMorph (ver. 1.2)^21^ to quantify the morphology of cells, actin, and nuclear DNA of haploid yeast cells. Of the 501 traits, 220 were coefficient of variation (CV) traits depending highly on the corresponding mean traits^50^. To prepare primarily independent traits, we converted the CV trait into a noise trait that was independent from the mean trait^51^. The non-linear correlation was determined by locally weighted scatterplot smoothing regression with a smoothing coefficient of 0.2, based on 2731 samples containing 749 replicates of 3∆ and 1982 quadruple mutants. We also employed the same non-linear correlation for the noise traits of drug treatment data and the morphological data of eight morphological test strains (Y8835, Y13206, three 1∆ strains, and three 2∆ strains).

Normalizations and *Z*-value calculations of the morphological data were performed using a GLM^52^. We employed the gamlss package in R^53^ to implement a GLM. The probability distribution and link function for each trait followed the assumptions used in Yang et al.^24^.

For normalization of the morphological data of the 1982 quadruple mutants, one-way analysis of variance (ANOVA) was applied to 749 replicates of 3∆, because the morphological data of the 1982 mutants were taken every time using the 749 replicates of 3∆ over 152 days. We used an experimental date as an explanatory variable. The better-suited model was selected by the Akaike information criterion by applying one-way ANOVA with the same variance to the morphological data of 749 replicates of 3∆ to determine whether there was a difference depending on the date of measurement for each of the 501 traits; the results showed a difference for 233 traits. Morphological data of 1982 quadruple mutants were normalized using the mean and variance most likely estimated by the selected model and converted to *Z*-values by the Wald test statistic in a manner of the one-sample tests^54^.

To normalize the eight morphological test strains [Y8835, Y13206 (3∆), three 1∆ strains, and three 2∆ strains; *n* = 5 for each], the difference in strains was first used as an explanatory variable to assume equal variance. Next, 40 samples (8 strains × 5 biological replicates) were normalized based on the estimated mean and variance of the parent strain and converted to *Z*-values by the Wald test statistic.

To normalize 3∆ cells treated with chemicals, we extracted dose-dependent morphological changes in 30 samples (6 doses × 5 biological replicates). This was because we compared morphological signatures between chemical-treated cells and gene-deletion mutants, but not morphological change itself. For this purpose, a simple regression analysis was applied to each trait assuming homoscedasticity, with the dose of the drug as an explanatory variable. The effect of the drug was converted from the slope estimated by simple regression analysis to the *Z*-value by the Wald test statistic.

### Phenotypic analysis of the 3∆ strain

To detect morphological alteration in the 3∆ (*pdr1∆ pdr3∆ snq2∆*) strain, one-way ANOVA was conducted for 3∆ (*n* = 5), *snq2∆* (*n* = 5), and their parent strain Y8835^11^ (*n* = 5) after normalization. A one-way ANOVA model assuming a difference between the three strains and the null model assuming no difference were applied to the data, and the likelihood was maximized by the maximum likelihood estimation method. Based on a likelihood ratio test *p*-value < 0.005 assuming a chi-square distribution on the ratio of the two likelihoods, we detected a significant difference among the three strains in 27 of the 501 traits. To estimate the FDR due to the multiplicity of tests, the explanatory variables were randomized and the test was repeated 3000 times with the same threshold value, resulting in an FDR of 0.3. Of the 27 traits detected, 24 differed significantly from the parent strain in at least one of the two mutants (*snq2∆* and 3∆) in the Wald test at *p* < 0.005 (FDR = 0.02): *snq2∆* and 3∆ had 14 and 16 traits that differed significantly from the parent strain, respectively. To summarize the morphological features of 3∆, a successive PCA^55^ was applied to the 24 traits, resulting in the extraction of six representative morphological features. The morphological characteristics of 3∆ are visualized in Fig. 2b. The morphological phenotype of 3∆ was compared with the other seven strains (parent strain, three 1∆ strains, and three 2∆ strains) by converting each morphological datum into a *Z*-value and performing PCA, illustrating the mutual relationship in a two-dimensional morphological space (Fig. 2c).

To evaluate the drug susceptibility using morphological changes as indicators, the wild-type strain (*his3∆*) and 3∆ were treated with 10 and 8 mM HU (*n* = 5), respectively, or 2 μg/mL ECB (*n* = 5), or 100 ng/mL TMN (*n* = 5). One-way ANOVA was conducted for the chemically treated cells and untreated cells after applying a GLM^52^ implemented in the gamlss package of R^53^. The one-way ANOVA model assuming the difference and the null model assuming no difference were applied to the data, and the likelihood was maximized by the maximum likelihood estimation method. The likelihood ratio test (*p* < 0.05, Bonferroni correction; *n* = 3006) assuming a chi-square distribution of the ratio of the two likelihoods obtained was applied to the lmtest package in R^56^. The HU-treated cells revealed 24 and 65 traits, the ECB-treated cells revealed 31 and 77 traits, and the TMN treated cells revealed 9 traits and 74 traits in *his3∆* and 3∆, respectively (Fig. 3a). Next, the mean and variance values were estimated by one-way ANOVA, converting each trait to a *Z*-value by the Wald test statistic, and PCA was performed to obtain six PCs. The *Z*-value of each repeating sample was calculated based on the estimated mean and variance and mapped to the six PCs (Fig. 3c). Using the six mapped PC scores, the Euclidean distance was calculated from untreated to treated in an orthogonal six-dimensional space.

To test the difference in HMA between *his3∆* and 3∆ with a one-way ANOVA, a GLM^52^ assuming a gamma distribution in the gamlss package^53^ was applied. The one-way ANOVA model assuming the difference between the three strains and the null model assuming no difference was applied to the data, and the likelihood was maximized by the maximum likelihood estimation method. A likelihood ratio test (*p* < 0.01, after Bonferroni-correction; *n* = 3) assuming a chi-square distribution for the ratio of the two likelihoods was tested using the lmtest package^56^. Finally, morphological similarity was compared between the parent strain and 3∆. PCA was performed with 749 replicates of 3∆ to obtain 259 PCs covering a cumulative contribution of 99%. Drug-treated cells of the parent strain and 3∆ were mapped to this morphological space and the correlation coefficient was calculated with the PC scores. When the correlation coefficient is R and the number of PCs is *n*, the *t*-value is converted by the formula: *t* = *R* × sqrt ([*n* − 2]/[1 − *r*^2^]). The upper *p*-value was determined by the correlation test assuming a t-distribution of *n* − 2 degrees of freedom (Fig. 3d).

### Prediction of drug targets by morphological profiling

To evaluate the similarity between the dose-dependent morphological changes in the drug-treated cells and the morphological changes in the gene-deleted strains, the correlation coefficient was calculated. In this analysis, 499 of the 501 traits were used, excluding 2 traits (ACV103_A1B and ACV103_C) in which missing values were found in the data of the quadruple mutants. To extract independent components from the 499 traits, PCA was performed with 749 replicates of 3∆, and 259 components covering a cumulative contribution of 99% were extracted as independent morphological traits. Each morphological profile was evaluated by mapping the 1982 quadruple mutant strains, 749 replicates of 3∆, and the *Z*-value of drug treatment (HU, ECB, TMN, BTZ, BML, MMS, and poacidiene) to the 259 PCs. Finally, Pearson’s product moment correlation coefficient was calculated between the morphological profiles of the dose-dependent changes and the morphological profiles of the quadruple mutants. The correlation coefficient test was performed by estimating the *p*-value (FDR = 0.05) using the probability distribution obtained by converting the correlation coefficient between the morphological profiles after the *t*-value was approximated with a Gaussian distribution by using the qvalue package in R^57^ (lower *y*-axes of Figs. 4a and 6b, and Supplementary Fig. 1).

### Preparation of functional gene groups

To prepare a functional group of genes suitable for target prediction, a new group of functionally related genes was set up. First, 2093 gene ontology (GO) terms that annotated three or more of the 1982 genes and less than 200 genes of a genome were selected to reduce the multiplicity of the comparison in GO enrichment analysis. Of the 1982 genes, 1637 genes were annotated with one or more of the 2093 GO terms. The distance between genes was calculated using a Boolean matrix representing the presence or absence of annotations between the 1637 genes and 2093 GO terms. Hierarchical cluster analysis was performed by the complete linkage method based on the obtained distance matrix to obtain 151 gene groups having a distance of 0.99 or less. Of these, 135 functional groups were composed of two or more genes, and each group contained a total of 1486 smaller sets of groups, thus this was designated as the representative set of gene functional groups containing 1637 genes. In addition, the gene-deleted strains were arranged in the order of the tree diagram of this cluster analysis (*x*-axes of Fig. 4 and Supplementary Fig. 1).

To evaluate the enrichment of gene function, a likelihood ratio test was performed using 1486 gene functional groups. First, the correlation coefficient between the drug-treated cells and the quadruple mutants and the correlation coefficient between the drug-treated cells and 749 replicates of 3∆ were converted into *t*-values. Then, a likelihood ratio test between the mean *t*-values of the gene function groups and 3∆ was performed by one-way ANOVA assuming a Gaussian distribution (*p* < 0.05, Bonferroni correction, *n* = 1486) (upper *y*-axes of Figs. 4a and 6b and Supplementary Fig. 1).

For each gene functional group, the most similar GO term was selected and used as the representative GO term for that group. Fisher’s exact test (FDR = 0.05, qvalue package of R^57^) was used to detect significantly enriched GO terms in each of the 135 gene groups using 2093 GO annotations and 1637 genes. As a result, 1729 GO terms were detected in total. Similarly, Fisher’s exact test (FDR = 0.05, qvalue package of R) detected significantly enriched GO terms in each of the 1486 gene group using 1729 GO terms and 1637 genes (Supplementary Data 17). Of these, the GO term with the lowest *p*-value in each group was designated as the representative GO term of that group (upper *y*-axes of Figs. 4a and 6b and Supplementary Fig. 1).

### Reproducibility of morphological phenotypes in the hypersensitive background

Morphological similarities between quadruple mutants and single mutants were compared using the 1982 morphologically important non-essential genes present in both datasets. For the morphological changes in quadruple mutants, the morphological profile of the 259 PCs was used. For the morphological changes in single mutants, the morphological profile of the 499 traits mapped to the 259 PCs was used after dimensional reduction. Then, both Pearson’s product-moment correlation coefficients were calculated. To test the correlation coefficients, 81641 pairs of correlation coefficients between 749 replicates of 3∆ and 109 replicates of *his3∆* were converted to *t*-values, and the null distribution was estimated by approximating with the Gaussian distribution. From the estimated null distribution, the upper *p*-value was calculated from the correlation coefficient of each mutant strain, and the FDR was estimated using the qvalue package^57^. Out of 1982 pairs, 535 were detected at an FDR of 0.05 (Fig. 5b left and Supplementary Data 13). The density distributions of 1982 quadruple mutants and 81641 parent strains were drawn by Kernel density estimation assuming a Gaussian distribution (Fig. 5a).

Morphological abnormalities of the quadruple mutants and single mutants were calculated by the Euclidean distance. To determine the degree of morphological abnormality of the quadruple mutants, the morphological data of 749 replicates of 3∆ in 499 traits were converted to *Z*-values using a GLM, and PCA was employed to obtain 259 PCs covering a cumulative contribution rate of 99%. Next, a morphological profile was calculated by mapping the *Z*-values of the quadruple mutants to 259 PCs. The Euclidean distance from the center of the 749 replicates of 3∆ in the 259-dimensional orthogonal space was defined as the degree of morphological abnormality in each mutant strain. Similarly, to determine the degree of morphological abnormality of a single mutant strain, the morphological data of 109 replicates of *his3∆* in 499 traits were first converted to *Z*-values using a GLM, and PCA was employed to obtain 57 PCs covering a cumulative contribution rate of 80%. Next, a morphological profile was calculated by mapping the *Z*-value of the single mutant strain to 57 components, and the distance from the center of the 109 replicates of *his3∆ i*n the 57-dimensional orthogonal space was defined as the degree of morphological abnormality in each mutant strain. After calculating the morphological abnormality for the parental strains in a similar way, the null distribution was estimated by approximating the gamma distribution. Using the estimated null distribution, the upper *p*-values of the 1982 quadruple mutants and the single mutants were calculated, and the FDR was estimated by *p*-values of the 3964 mutant strains using the qvalue package in R^57^. In this way, we identified 321 of 1982 genes that significantly impacted morphological abnormality in both quadruple and single mutants (FDR = 0.001) (Fig. 5b, right). Of these, 222 mutants had significantly similar morphologies (FDR = 0.05) (Fig. 5b right and Supplementary Data 13).

We investigated whether the gene functions affected in the single mutants were also affected in the quadruple mutants for 1982 non-essential genes. For this purpose, the degrees of morphological abnormality of gene-deleted strains in the 135 gene groups were compared. The morphological abnormalities of each of the single mutants and the quadruple mutants were calculated as described above and used to perform a one-way ANOVA with a GLM model^52^ assuming a gamma distribution with the gamlss package in R^53^. Using 152 gene groups as explanatory variables, one-way ANOVA with a different variance for each group was performed separately for quadruple mutants and single mutants. After multiplicity was estimated using the qvalue package^57^ for the upper *p*-values of 270 groups of quadruple mutants and single mutants, the Wald test was performed to assess whether the morphological abnormalities of the 135 functional groups composed of two or more genes were significantly greater than 3∆ and *his3∆* in the quadruple mutants and single mutants, respectively (FDR = 0.01) (Fig. 5c). Our analysis indicated that 129 and 134 gene groups were detected in the quadruple mutants and single mutants, respectively, with 128 gene groups in both (Fig. 5d).

### Antifungal susceptibility test

Antifungal susceptibility against *S. cerevisiae* wild-type and deletion mutants was tested in the drug hypersensitive genetic background 3∆ (*pdr1∆ pdr3∆ snq2∆*). Yeast cells were grown in YPD at 30 °C with shaking at 200 rpm overnight to reach a logarithmic phase (1–5 × 10^7^ cells/mL). Overnight cultures were diluted with YPD and then inoculated in YPD containing 3% DMSO (with and without drugs) to reach 1–5 × 10^5^ cells/mL and incubated at 30 °C in a static incubator. Poacidiene and poacic acid concentrations ranged from 0 to 256 μg/mL, and from 0 to 1024 μg/mL, respectively. After 18 h of incubation in 96-well flat-bottom microtiter plates (Corning Inc., Corning, NY, USA), the cell suspension was stirred with a Titramax 1000 rotator (Heidolph, Schwabach, Germany). The optical density of wells was measured using a SpectraMax Plus 384 plate reader (Molecular Devices, San Jose, CA, USA) at 600 nm. The inhibitory concentration of minimal inhibitory concentration plates at 50% cell survival (IC_50_) was calculated from dose-response curves. The IC_50_ was estimated using Markov chain Monte Carlo methods (5000 iterations including first 2000 iterations as warm-up in eight chains each) with the rstan package (https://mc-stan.org/users/interfaces/rstan) using reparameterizing of the four-parameter log-logistic equation implemented in the drc package in R^58^. 3∆ and quadruple mutants (*mms1∆, mms22∆, rtt101∆, rtt107∆, rtt109∆, ctf18∆, rad51∆, rad52∆, rad54∆, rdh54∆*, and *srs2∆*) were also tested for poacidiene-sensitivity ranging from 0 to 100 μg/mL. To test whether the IC_50_ values of the quadruple mutants differed significantly from 3∆, we employed a likelihood ratio test between the full and null models. The models indicated differences among 3∆ and quadruple mutants (*mms1∆, mms22∆, rtt101∆, rtt107∆, rtt109∆, ctf18∆, rad51∆, rad52∆, rad54∆, rdh54∆*, or *srs2∆*) in all of the four parameters of the log-logistic equation and differences other than IC_50_ values between 3∆ and each quadruple mutant, respectively. Thus, the difference in the degree of freedom between the full model and each null model was one. The *p*-value was calculated using the chi-squared distribution when the upper tail of IC_50_ was lower than 3∆ or the lower tail of IC_50_ was higher than 3∆ after Bonferroni correction (*n* = 11).

### Antifungal assays of poacidiene against phytopathogenic fungi

The antifungal effects of poacidiene on the colony growth of *R. solani* and *P. aphanidermatum* were examined by the agar dilution method. Briefly, the antifungal activity against these fungi was evaluated at 50, 100, 200, 300 µg/mL poacidiene. Actively growing fungi plugs (2 mm in diameter) were obtained and placed at the center of Petri dishes in potato-dextrose-agar (PDA) culture medium with poacidiene dissolved in DMSO. PDA plates containing DMSO were used as control plates. The cultures were incubated at 25 °C and the radial growth of mycelia was measured (*n* = 3) after 24 h for *R. solani* and after 15 h for *P. aphanidermatum*. The percentage inhibition was calculated compared with the growth on the control plates.

The antifungal effects of poacidiene on the colony growth of *P. sojae*, *A. alternata* and *A. solani* were also examined using the agar-dilution method, but in a slightly different way. *P. sojae* was grown on corn meal, while *A. alternata* and *A. solani* field strains were grown on PDA at room temperature. The growth inhibition of poacidiene on the respective solid agar cultures was assessed by generating replicate plates (*n* = 3) at 0, 500, 1000, and 1500 µg/mL poacidiene. The plates were inoculated with an actively growing plug of *P. sojae*, *A. alternata* or *A. Alternata* grown at room temperature. The diameter of the plug used for inoculation was 5 mm. The diameter of hyphal growth (*A. alternata* and *A. solani*) was measured after 120 h of incubation.

### Synthesis of poacidiene

The poacidiene (8-8dc diferulic acid, dc = decarboxylated) required for this study was prepared by unoptimized treatment of ferulic acid dilactone with concentrated ammonia (28–30% NH_3_ in water) at 50 °C overnight as shown in Scheme ﻿1. Ferulic acid dilactone was made from ferulic acid by peroxidase-catalyzed free-radical coupling in aqueous acetone (20% acetone v/v) using the hydrogen peroxide-urea complex as an oxidant.

**Scheme 1:**
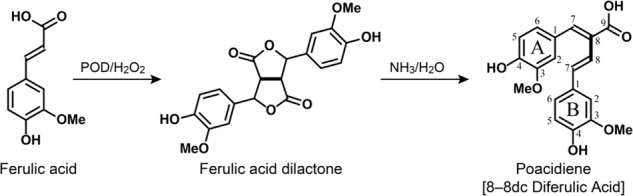
Synthetic scheme for producing poacidiene. The bolded bond is that formed during radical coupling (and indicate why this is termed an 8–8 diferulate-derived compound). The ring designations (A and B) and numbering are for NMR assignment purposes (Supplementary Figs. 2–4).

#### Preparation of ferulic acid dilactone

We essentially followed Ralph et al.^33^ and He et al.^34^ for the dilactone preparation. Briefly, 19.4 g ferulic acid was dissolved in 400 mL of acetone. The solution was then diluted by adding 1500 mL of water, followed by adding 5.7 g of H_2_O_2_-urea dissolved in 80 mL water. Finally, the coupling reaction was initiated by adding 40 mg of horseradish peroxidase dissolved in 20 mL water while the reaction solution was well stirred. The reaction was kept for 60 min. After acidification of the reaction mixture (pH < 3), the precipitated products were recovered by filtration and air-dried. The crude products (15 g, 77%) were purified by crystallization from ethyl acetate producing the dilactone product (4.5 g, yellow solid, 22% yield); NMR data (Supplementary Figs. 2–4) were consistent with those in the literature^33,34^.

#### Preparation of poacidiene

FA-dilactone (3.0 g) was dissolved in 20 mL of concentrated ammonia in a 25 mL vial fitted with a screw cap and a Teflon liner. The solution was heated at 50 °C in a sand bath overnight (16–18 h). After cooling to room temperature, the reaction mixture was transferred dropwise into 4 M HCl solution (200 mL). The products were recovered by ethyl acetate extraction (200 mL × 2). The insoluble products (between layers, mainly 8-8o diferulic acid, the opened form) were filtered, the ethyl acetate phase was dried over MgSO_4_ and evaporated under reducing pressure on a rotatory evaporator. The residues were dissolved (suspended) in 30 mL ethyl acetate, the insoluble solid (8-8o diFA) was filtered and washed with a small amount of ethyl acetate. The combined ethyl acetate solution was concentrated to about 20 mL and loaded onto a silica-gel column (100 g silica, Biotage SNAP column) eluting with hexanes/EtOAc (1/1, v/v) containing 1% acetic acid. A fraction containing the target product poacidiene was collected, from which the product was crystalized from ethyl acetate/hexanes to obtain a yellow solid (0.6 g, mainly *trans*-configuration, 20% yield). Textual NMR data for poacidiene are provided in NMR Spectra section.

### NMR spectra

NMR spectra of the synthetic poacidiene standard in acetone-d_6_ were acquired on a Bruker Avance III 500 MHz spectrometer (Bruker Biospin, Bruker, Billerica, MA, U.S.A.) equipped with a cryogenically cooled 5 mm ^1^H/^13^C-optimized triple resonance (^1^H/^13^C/^15^N) TCI gradient probe with inverse geometry (proton coils closest to the sample). Full assignments were unambiguously made using the usual array of 1D and 2D (including HSQC, HMBC, and COSY) experiments. The 1D proton and carbon NMR spectra and the 2D HSQC are provided here, Figs. S2–4, and full original datasets in Bruker’s Topspin format are available upon request (jralph@wisc.edu).

#### Textual NMR data for poacidiene

NMR (500 MHz, acetone-d_6_) δ_H_: 7.54 (1H, s, A7), 7.24 (1H, d, *J* = 16.4 Hz, B7), 7.19 (1H, d, *J* = 2.0 Hz, A2), 7.13 (1H, d, *J* = 2.0 Hz, B2), 7.06 (1H, ddd, *J* = 8.2, 2.0, 0.7 Hz, A6), 7.04 (1H, dd, *J* = 16.4, 1.1 Hz, B8), 6.98 (1H, ddd, *J* = 8.2, 2., 0.6 Hz, B6), 6.91 (1H, d, *J* = 8.2, A5), 6.81 (1H, d, *J* = 8.1, B5), 3.86 (3H, s, B3-OMe), 3.8419 (3H, s, A3-OMe); δ_C_: 168.84 (A9), 148.58 (A4), 148.51 (B3), 148.15 (A3), 147.71 (B4), 139.10 (A7), 135.18 (B7), 130.71 (B1), 128.44 (A1), 128.24 (B8), 125.29 (A6), 120.88 (B6), 120.53 (A8), 116.03 (B5), 115.95 (A5), 114.30 (A2), 110.13 (B2), 56.16 (B3-OMe), 56.12 (A3-OMe).

## Supplementary information

Supplementary Figures

Supplementary Data 1

Supplementary Data 2

Supplementary Data 3

Supplementary Data 4

Supplementary Data 5

Supplementary Data 6

Supplementary Data 7

Supplementary Data 8

Supplementary Data 9

Supplementary Data 10

Supplementary Data 11

Supplementary Data 12

Supplementary Data 13

Supplementary Data 14

Supplementary Data 15

Supplementary Data 16

Supplementary Data 17

## Data Availability

All data provided in this study are available without restriction upon request. Full microscopy image data sets are available in SCMD (*Saccharomyces cerevisiae* morphological database, http://www.yeast.ib.k.u-tokyo.ac.jp/SCMD/summary.php?pj=quadruple).

## Code availability

The codes used for the computational analyses are available upon request to the authors.
