# Supplementary material for: High-throughput platform for yeast morphological profiling predicts the targets of bioactive compounds
Source: NPJ Syst Biol Appl. 2022 Jan 27;8:3. doi: 10.1038/s41540-022-00212-1 (PMC8795194; doi:10.1038/s41540-022-00212-1)
Supplement: Supplementary file 1 — Supplementary Figures [file 41540_2022_212_MOESM1_ESM.pdf]

Supplementary Information

High-throughput platform for yeast morphological profiling predicts the targets of bioactive compounds

Shinsuke Ohnuki, Itsuki Ogawa, Kaori Itto-Nakama, Fachuang Lu, Ashish Ranjan, Mehdi Kabbage, Abraham Abera Gebre, Masao Yamashita, Sheena C. Li, Yoko Yashiroda, Satoshi Yoshida, Takeo Usui, Jeff S. Piotrowski, Brenda J. Andrews, Charles Boone, Grant W. Brown, John Ralph, Yoshikazu Ohya

Supplementary Figure 1. Morphological profiling of drugs with known targets.
Supplementary Figure 2. Proton NMR spectra of poacidiene.
Supplementary Figure 3. Carbon NMR spectra of poacidiene.
Supplementary Figure 4. HSQC spectrum of poacidiene.
Supplementary Figure 5. Effects of poacidiene on the growth of phytopathogenic fungi.

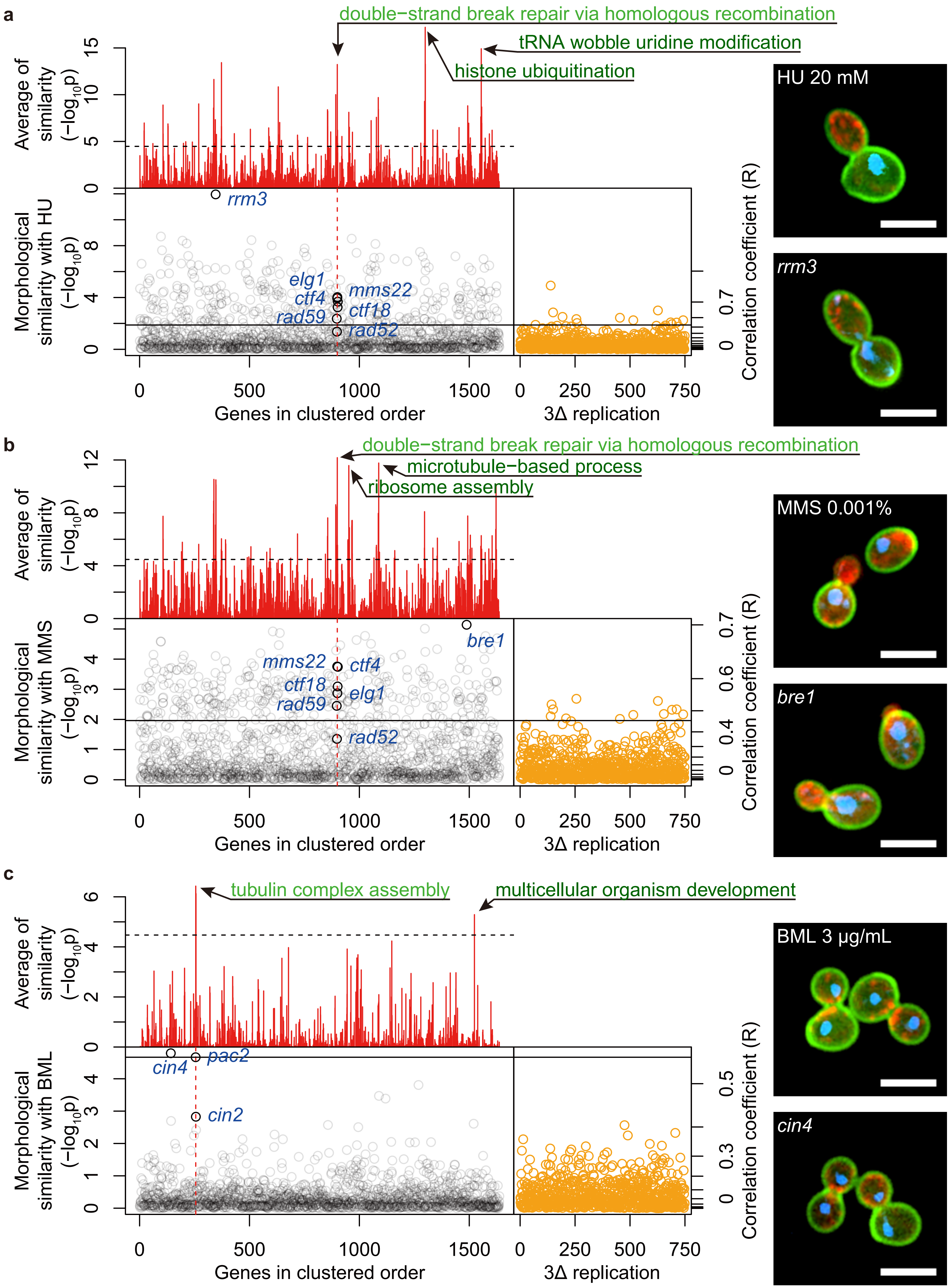
a
double−strand break repair via homologous recombination
tRNA wobble uridine modification
histone ubiquitination
Average of similarity (−log10p)
Morphological similarity with HU (−log10p)
rrm3
elg1
ctf4
rad59
mms22
ctf18
rad52
Genes in clustered order
3Δ replication
Correlation coefficient (R)
HU 20 mM
rrm3
b
double−strand break repair via homologous recombination
microtubule−based process
ribosome assembly
Average of similarity (−log10p)
Morphological similarity with MMS (−log10p)
bre1
mms22
ctf4
ctf18
elg1
rad59
rad52
Genes in clustered order
3Δ replication
Correlation coefficient (R)
MMS 0.001%
bre1
c
tubulin complex assembly
multicellular organism development
Average of similarity (−log10p)
Morphological similarity with BML (−log10p)
cin4
pac2
cin2
Genes in clustered order
3Δ replication
Correlation coefficient (R)
BML 3 μg/mL
cin4

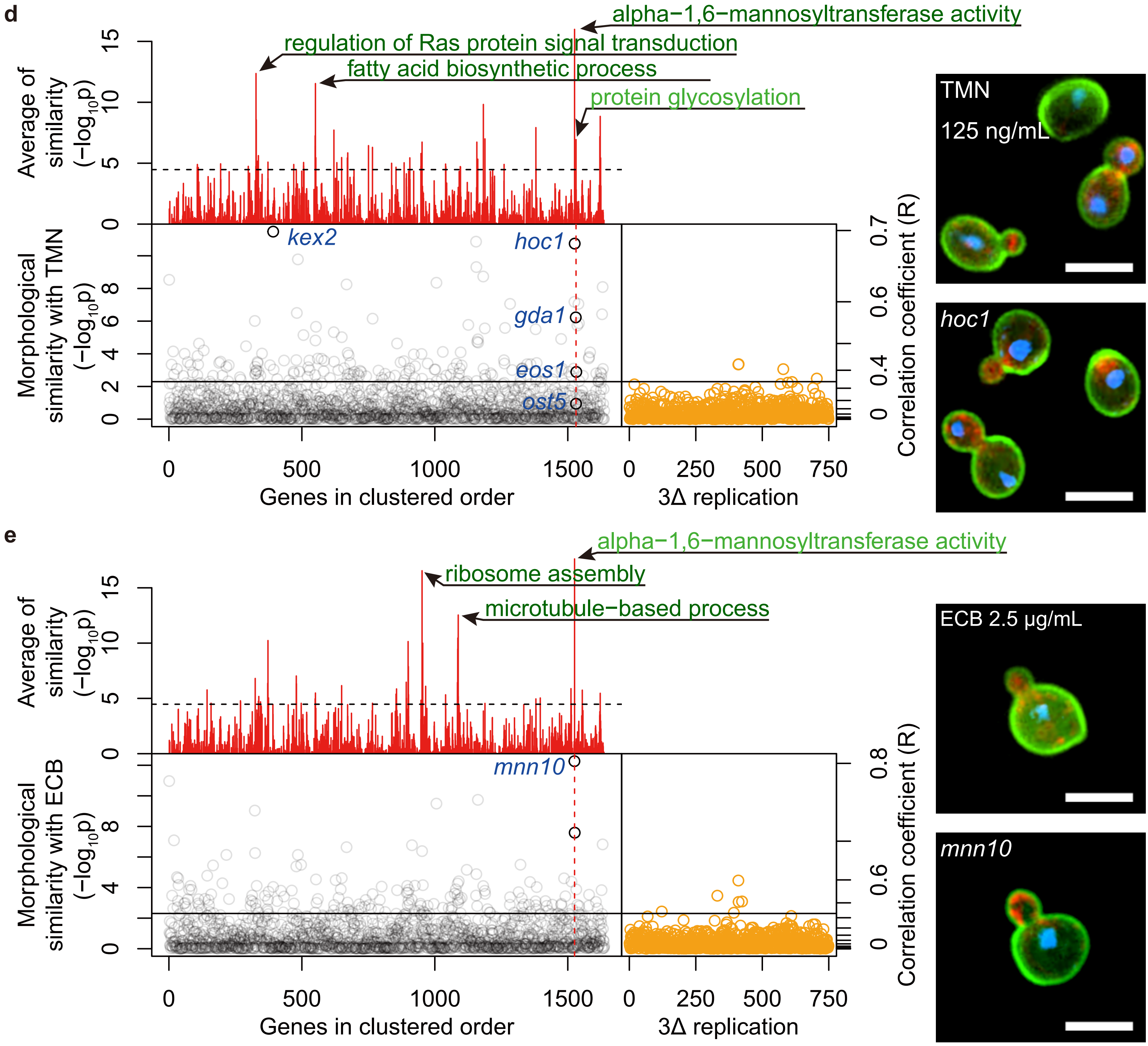

Supplementary Figure 1. Morphological profiling of drugs with known targets.
(a) HU. (b) MMS. (c) BML. (d) TMN. (e) ECB. Legends are the same as in Figure 4a. Enriched GOs in the detected gene groups ($p < 0.05$ after the Bonferroni correction) for HU, MMS, BML, TMN, and ECB are listed in Supplementary Data 8, Data 9, Data 10, Data 11, and Data 12, respectively. Significantly similar mutants (FDR = 0.05) for HU, MMS, BML, TMN, and ECB are listed in Supplementary Data 3, Data 4, Data 5, Data 6, and Data 7, respectively.

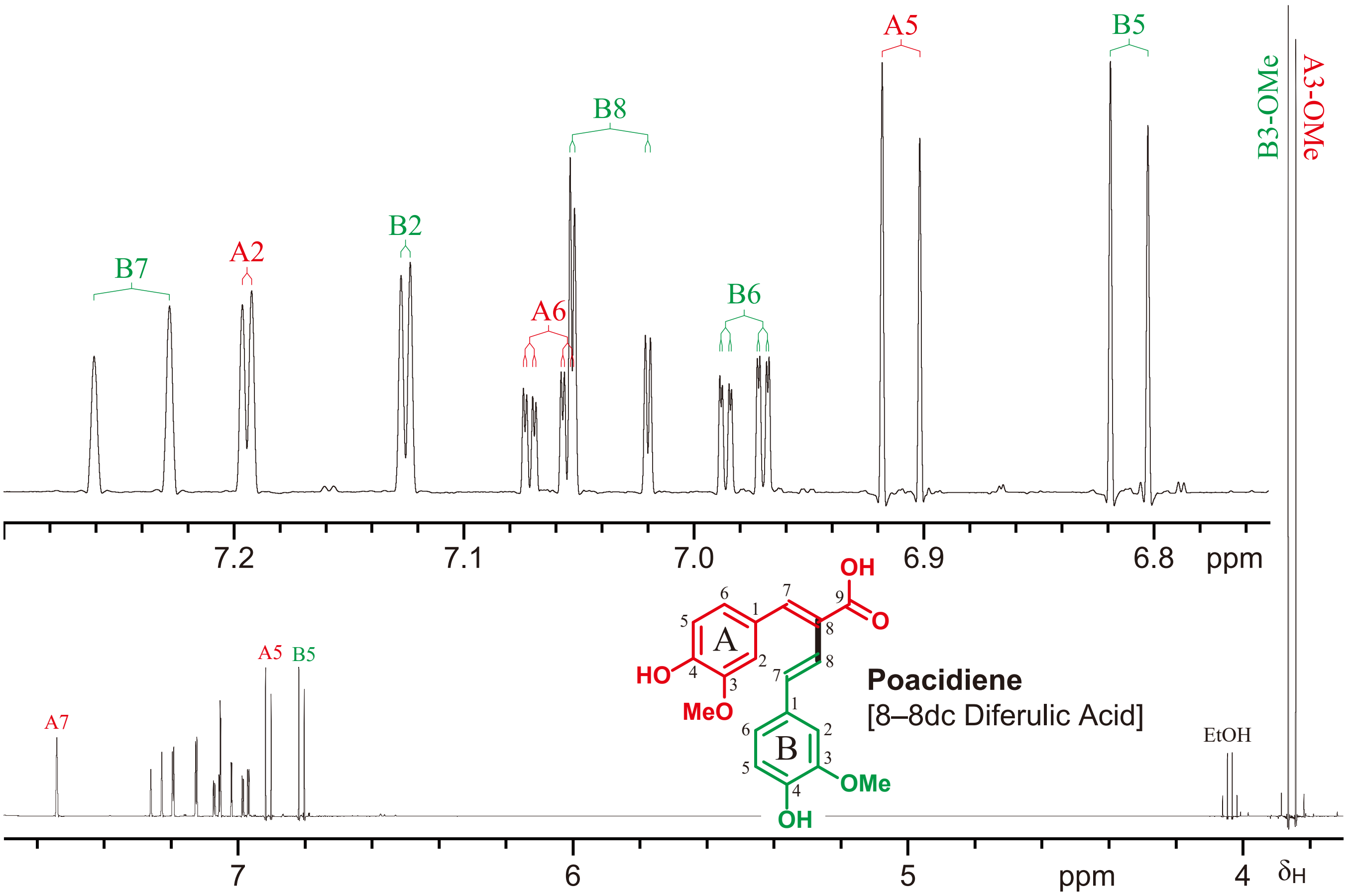

Supplementary Figure 2. Proton NMR spectra of poacidiene.

Color coding: red for the A-ring moiety, green for the B-ring moiety.

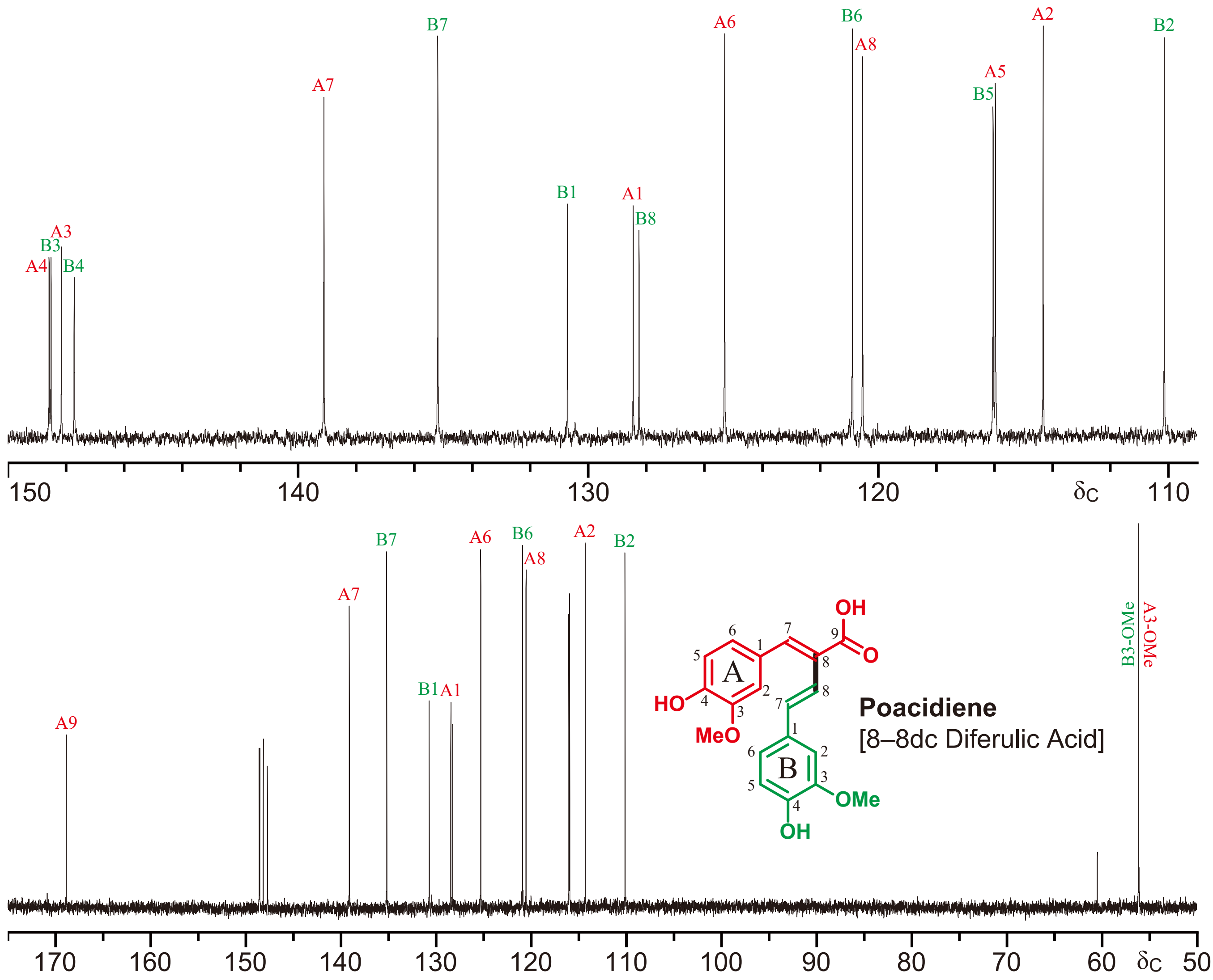

Supplementary Figure 3. Carbon NMR spectra of poacidiene.

Color coding: red for the A-ring moiety, green for the B-ring moiety.

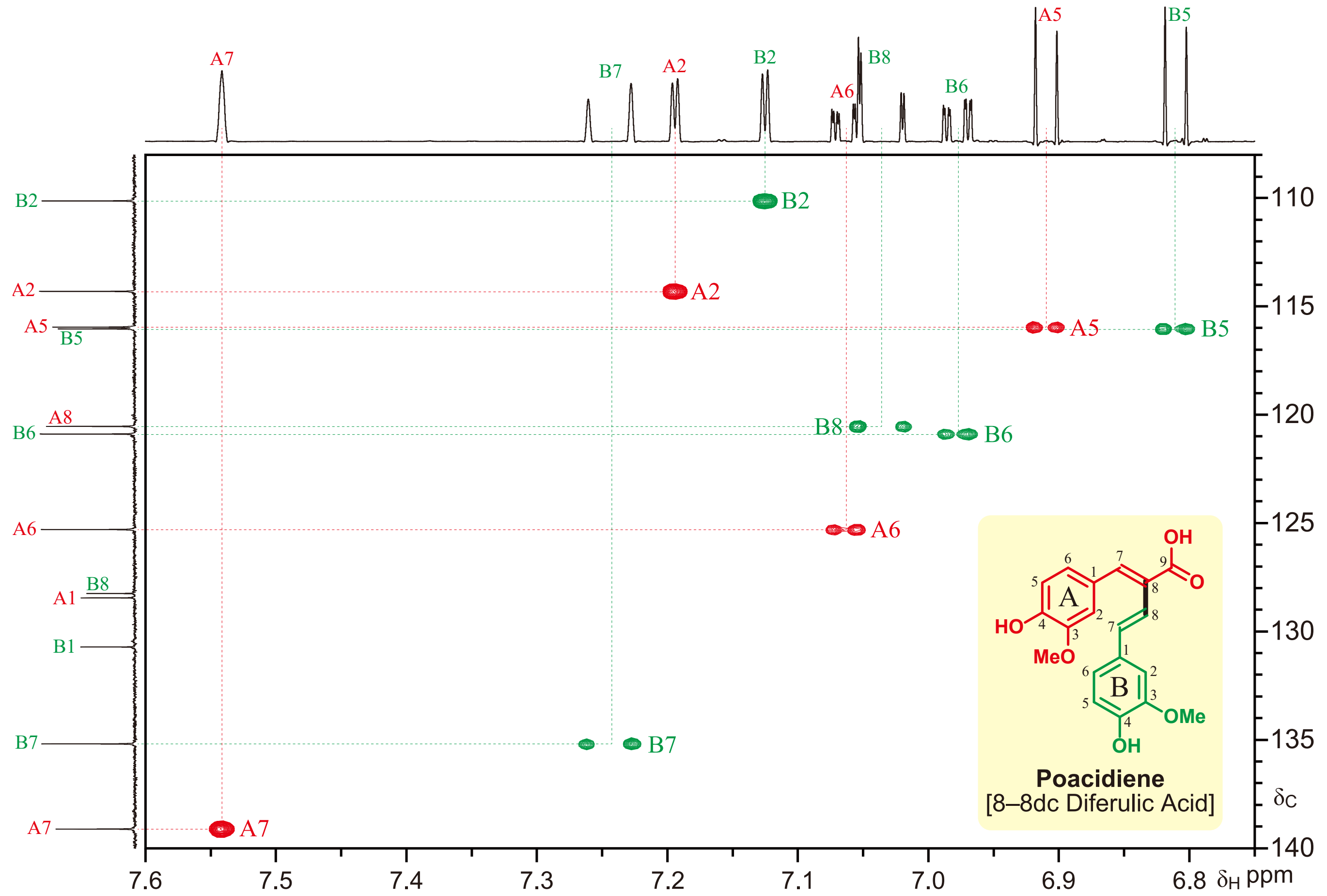

Supplementary Figure 4. HSQC spectrum of poacidiene

Obviously, correlation from the methoxyl and A7 groups are also present (not shown), producing the data noted in the text above (and in the main paper). Color coding: red for the A-ring moiety, green for the B-ring moiety.

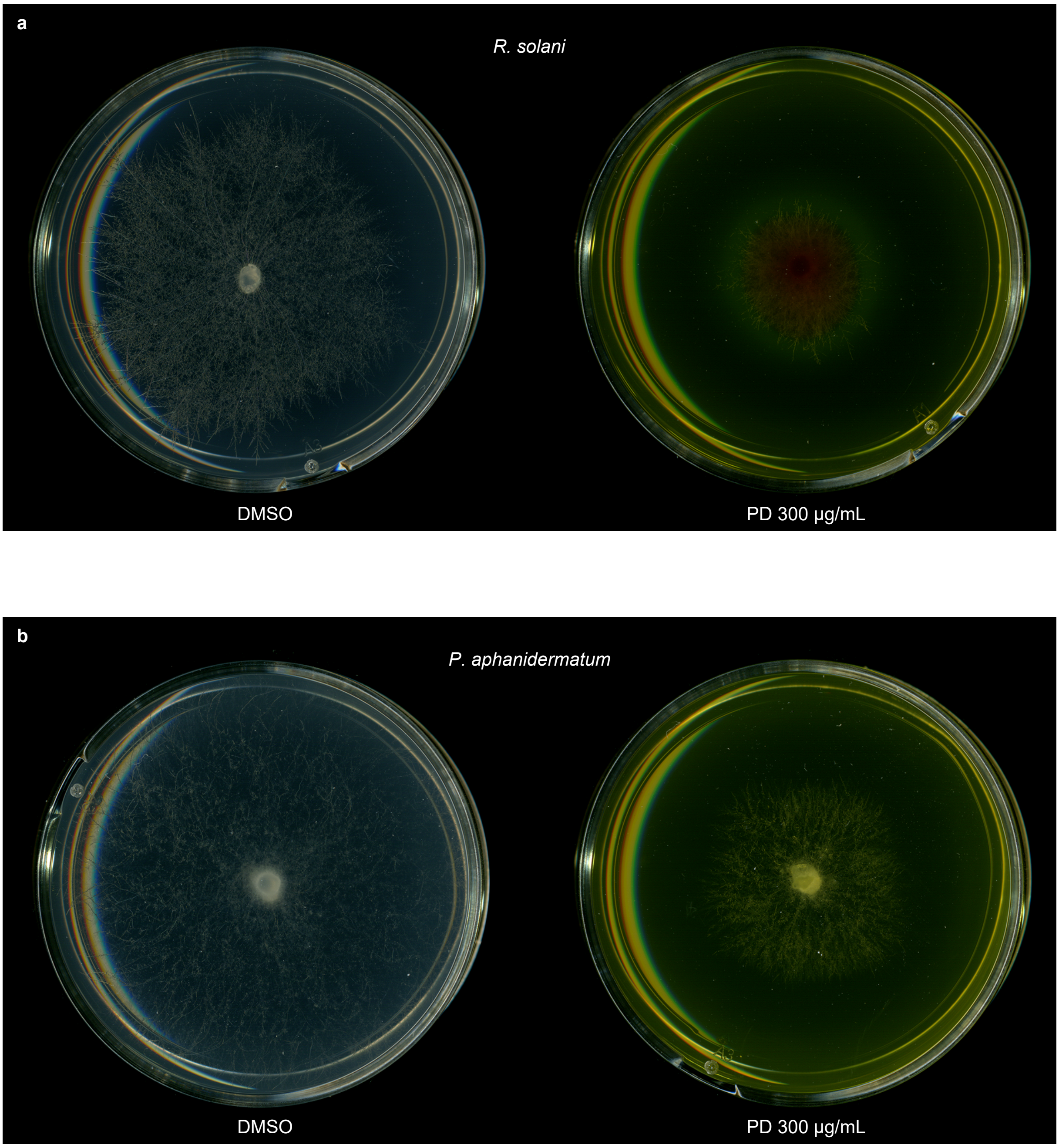

Supplementary Figure 5. Effects of poacidiene on the growth of phytopathogenic fungi.
Photographs of (a) the filamentous fungi *R. solani* and (b) the oomycete *P. aphanidermatum* grown on the PDA plates with/without 300 µg/mL of poacidiene. Photographs are shown without enhancement of the contrast. Contrast-enhanced photographs are shown in Figures 8a and 9a.
